# Precision of Radiation Chemistry Networks: Playing Jenga with Kinetic Models for Liquid-Phase Electron Microscopy

**DOI:** 10.1021/prechem.3c00078

**Published:** 2023-12-06

**Authors:** Birk Fritsch, Paolo Malgaretti, Jens Harting, Karl J. J. Mayrhofer, Andreas Hutzler

**Affiliations:** † Helmholtz Institute Erlangen-Nürnberg for Renewable Energy (IEK-11), Forschungszentrum Jülich GmbH, Cauerstr. 1, 91058 Erlangen, Germany; ‡ Department of Chemical and Biological Engineering and Department of Physics, Friedrich-Alexander-Universität Erlangen-Nürnberg, Cauerstr. 1, 91058 Erlangen, Germany

**Keywords:** Electron beam effects, gold, radiolysis, kinetic modeling, simulation efficiency, liquid cell transmission electron microscopy

## Abstract

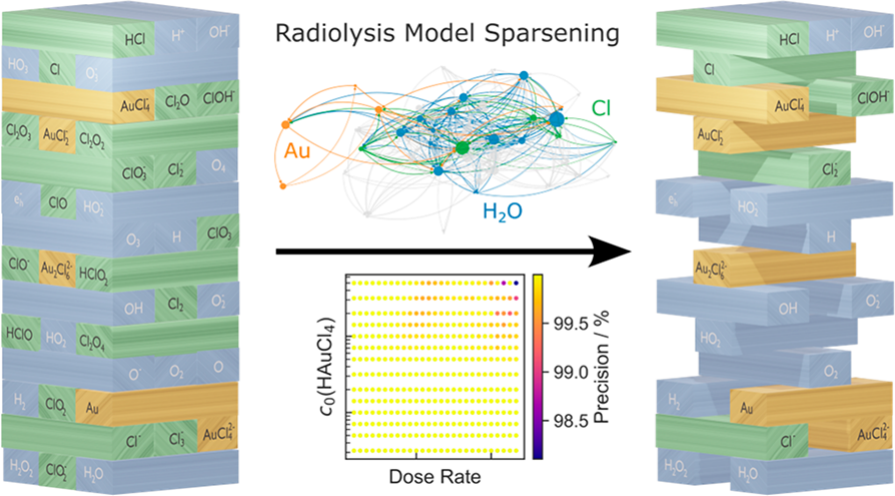

Liquid-phase transmission electron microscopy (LP-TEM) is a powerful tool to gain unique insights into dynamics at the nanoscale. The electron probe, however, can induce significant beam effects that often alter observed phenomena such as radiolysis of the aqueous phase. The magnitude of beam-induced radiolysis can be assessed by means of radiation chemistry simulations potentially enabling quantitative application of LP-TEM. Unfortunately, the computational cost of these simulations scales with the amount of reactants regarded. To minimize the computational cost, while maintaining accurate predictions, we optimize the parameter space for the solution chemistry of aqueous systems in general and for diluted HAuCl_4_ solutions in particular. Our results indicate that sparsened kinetic models can accurately describe steady-state formation during LP-TEM and provide a handy prerequisite for efficient multidimensional modeling. We emphasize that the demonstrated workflow can be easily generalized to any kinetic model involving multiple reaction pathways.

## 1 Introduction

*In situ* and *operando* studies have been demonstrated as outstanding instruments for investigating dynamics in real time on site. This is particularly relevant if processes of interest are occurring on the nanoscale. Here, access is granted by employing X-rays or high energy electrons. In particular liquid-phase transmission electron microscopy (LP-TEM) enables unique insights into multifarious fields of applied research, such as nanochemistry,^1,2^ energy conversion,^3^ or geoscience,^4,5^ down to atomic resolution.^6^ Yet, accurate interpretation of such studies is nontrivial and often relies on simulations.

Simulations facilitate gaining crucial insights and detecting causalities that may not be directly accessible experimentally. However, this is often accompanied by high computational effort that relates to notable energy consumption and runtime.^7^ This is especially relevant for multidimensional time-dependent modeling. As computation has become a powerful tool to complement cutting-edge research in the landscape of *in situ* and *operando* studies,^8^ cost-efficient models are desirable for routinizing this synergetic approach.

Here, simulations are particularly relevant to estimate artificially introduced effects triggered, e.g., by high energy irradiation. However, the required kinetic models are not yet optimized toward cost-effectiveness. Furthermore, it remains unclear at which point a chemical reaction network is complete, comprising all relevant reaction paths to reflect the experimental conditions.

Exposing aqueous systems to ionizing radiation triggers a physicochemical reaction cascade that causes the formation of so-called primary products (e_h_^–^ denotes solvated electrons).^9^ For low linear energy transfer (LET) irradiation, such as electrons, the following primary products are formed:
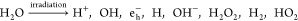
1After about 1 μs, those are equilibrated so that this reaction cascade can be summarized by radiation-specific generation values (G-values, *G*_*i*_).^9^ Solution chemistry (and when relevant diffusion) must be considered to account for the subsequent reactions of the solvent molecules with primary species. This requires a comprehensive reaction network. To track the changes in concentration *c* of a chemical species *i* over time *t*, radiation chemistry simulations are performed by solving a set of coupled differential equations that represent the kinetic interplay within the chosen network:
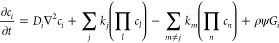
2Here, *D* is the diffusion coefficient and *k* is the respective rate constant of reactions involving *l* species where *i* is a reaction product, or *n* species where *i* is serving as an educt. The density of the medium is described by *ρ*, whereas *ψ* denotes the dose rate. A comprehensive derivation of this approach is described elsewhere.^10^

The order of this reaction matrix (eq 2), and thus, the computational cost is directly related to the amount of chemicals involved. Particularly when heterogeneous geometry-dependent systems are simulated, relatively quick approaches that neglect diffusion by assuming a homogeneous volume are often only coarse approximations. Instead, meshed finite-element approaches are required. As a consequence, this cost can scale up drastically. Such simulations are, for instance, relevant for addressing fundamental effects within the nm-scaled nano reactor such as confinement-driven concentration alterations,^11^ liquid–gas transitions,^12^ beam-induced electric fields,^12^ accurate description of liquid flow fields and mixing,^13,14^ as well as secondary electron emission caused by solid specimen themselves^15^ or electrodes within the field of view during *operando* electrochemistry studies.^16,17^ This becomes especially relevant for modeling of LP-TEM experiments in scanning mode (STEM),^18^ where the beam typically rasters over several spatial positions whose number can scale up easily.

In the context of LP-TEM, most reaction chemistry simulations are based on the reaction set by Elliot and McCracken^19^ which was adapted to LP-TEM by Schneider et al.^10^ The reaction set consists of 16 chemicals. Recently, the set was appended by para-oxygen (O),^20^ to account for additional reaction pathways. Likewise, particularly temperature-dependent modeling in the context of LP-TEM has been performed using a less comprehensive reaction set.^21−23^ Introduced by Elliot and Bartels,^24^ the latter consists of only 12 reactants. To date, no evaluation of the interchangeability of these sets has been performed in the context of LP-TEM.

Reaction sets are usually expanded by relevant groups of chemicals to account for the radiation-triggered interplay with solutes. Again, the amount of species per group can differ drastically in the literature, even for similar systems. For instance, the amount of additional species for the simulation of solutions containing chloro-gold complexes, arguably one of the most frequently studied model systems in LP-TEM, differ substantially from two^21^ up to 25^20^.

Naturally, this coincides with an increase in the predictive power of the model. But does this really require such a high increase in dimensionality? To answer this question, we define a set of parameters that are experimentally accessible, first for the most general case, pure water, and second for aqueous solutions of HAuCl_4_, of which literature provides one of the most diverse interpretations in modeling,^15,20,21,25,26^ and to date the most comprehensive kinetic model applied to LP-TEM.^20^ By subsequently sparsening the respective reaction sets, we unveil a minimum set of species required for reliable, aqueous radiation chemistry simulations in LP-TEM.

Our findings are crucial to perform resource-effective simulations of radiation chemistry during *in situ* and *operando* studies of aqueous solutions. Albeit presented on the use case of LP-TEM, this also applies to X-ray-based *operando* investigations, which are prone to radiation-caused alterations.^27−29^

## 2 Methods

Radiation-chemistry modeling is performed using AuRaCh, a Python-based tool introduced earlier.^20^ All simulations are conducted by using a homogeneous voxel approximation. Thus, the diffusion term in eq 2 is omitted, leaving a set of ordinary differential equations (ODEs):
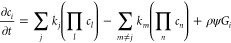
3In all simulations, we assume aerated water with an O_2_ concentration of 0.255 mM^10^. For HAuCl_4_ solutions, electron-beam interaction with the aqueous matrix alone is considered via the *G*_*i*_ given in Table 1. The tabular representations of the kinetic models can be found in Table S1 of the Supporting Information.

**Table 1:** Generation Values Used in This Work^10,46^

reactant	e_h_^–^	H^+^	OH^–^	H_2_O_2_	H	OH	HO_2_	H_2_	H_2_O
*G*_*I*_ / (molecules/100 eV)	3.47	4.42	0.95	0.47	1.00	3.63	0.08	0.17	–5.68

Conversion from the electron-flux density *ϕ* to dose rate was done using the following formula:^22^
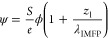
4Here, *S* is the stopping power, *e* is the elementary charge, λ_IMFP_ is the inelastic mean free path in water, and *z*_l_ is the liquid thickness. To do so, an acceleration voltage of 300 kV and a liquid thickness of 100 nm is assumed. Thus, *S* amounts to 2.36 (30) and λ_IMFP_ to 380 nm^31^.

A script to sparse reaction files for the usage with AuRaCh is available under https://github.com/BirkFritsch.

## 3 Results

In this study, we aim at studying the eventually yielded steady-state concentrations, as they are typically interpreted in case of homogeneous voxel simulations for LP-TEM. At dose rates relevant to LP-TEM, those are formed within a fraction of a second.^10,32^ Note, however, that the transient period can extend up to several hours at low dose rates.^20,32^

To reduce the parameter landscape within radiation chemistry simulations in LP-TEM, first the basis of all aqueous solutions is considered. By taking a comprehensive reaction set, we start with 17 reactants, namely H_2_O, H^+^, OH^–^, H_2_, O_2_, H, OH, H_2_O_2_, HO_2_, HO_2_^–^, HO_3_, O, O^–^, O_2_^–^, O_3_, O_3_^–^, and e_h_^–^. These species interact via 86 chemical reactions (see Supporting Table S1 for details). In order to sparse the reaction set and hence reduce the dimensionality of the resulting ODE system (see again eq 3) the contribution of a reactant *i* can be disabled by setting the rate constant of all reactions involving *i* to zero. To maintain the applicability of a sparsened reaction network, we define the following constraints:

- First, any reactants that are present prior to irradiation must be maintained within the model. Practically speaking, this concerns all reactants with nonzero initial concentrations. This concerns H_2_O, O_2_, H^+^ and OH^–^.
- Second, primary species (see again eq 1) must remain within the model, as their exclusion would violate mass and/or charge balance.
- Third, the steady state concentrations of the solvent and of the main products should remain unchanged.

As main products, we consider relatively stable chemicals (e.g., H_2_O_2_ decomposes slowly) that are experimentally accessible *in situ*, e.g., via electron energy loss spectroscopy^33^ or electrochemical approaches.^34^ For pure water, this denotes H_2_, H_2_O_2_, and O_2_. Moreover, we track the parameters defining the acidity under irradiation, namely the radiolytic acidity π* and the radiolytic ion product *K*_W_^*^.^5,32^ Those are defined by
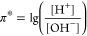
5
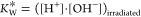
6Thus, we implicitly track the concentrations of H^+^ and OH^–^, as well. We regard any of these parameters as reasonably unchanged if the relative change between the outcome of the complete, initial model and the model with the reduced reaction set remains within a 5% margin.

For pure water, the defined constraints leave HO_2_^–^, O_2_^–^, HO_3_, O_3_, O_3_^–^, and O^–^ as possibly expendable candidates. To gauge the relevance of these residual species, we performed systematic spot check simulations within the parameter space of different initial pH (although the interpretability of pH becomes questionable under high intensity irradiation, the initial concentrations of H^+^ and OH^–^ still affect the radiation chemistry^32^) and dose rate.

Eliminating HO_2_^–^ or O_2_^–^ immediately causes simulated steady-state concentrations to surpass almost all of our tolerance limits (see Supporting Figures S1 and S2), particularly at higher dose rates relevant to LP-TEM. Hence, these species are regarded as essential within the kinetic model.

**Figure 1:**
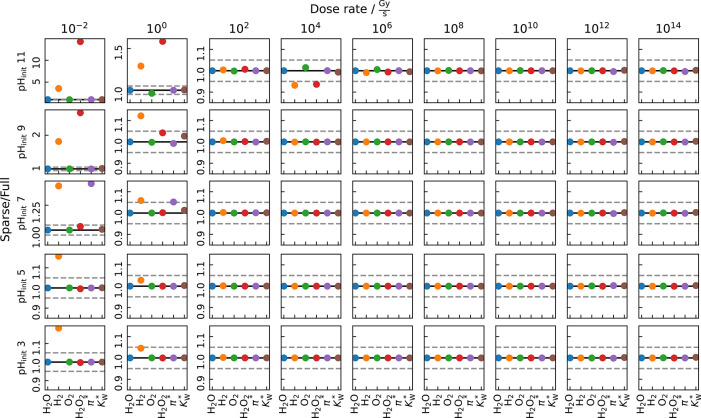
Spot checks on the relative deviation of the testing parameters of pure water after disabling HO_3_, O_3_, and O_3_^–^. The solid line denotes a perfect agreement between the full and sparsened models, whereas the dashed lines mark the 5% accuracy limit.

On the other hand, disabling either one, or even all O_3_-related candidates (HO_3_, O_3_, O_3_^–^) has little to no effect on the precision of the defined testing parameters (see Figure 1). Only at low dose rates smaller than or equal to 1 Gy/s are drastic deviations visible. Remarkably, those appear to increase with initial pH. Moreover, slight changes of the H_2_ and H_2_O_2_ accuracy are eminent for an initial pH 11 at 10 kGy/s. However, even this high dose rate is barely accessible during typical LP-TEM experiments.

Excluding O^–^ provides little to no violations within the spot-checked parameter space. Only at low dose rates at an initial pH of 11 slight variations in the steady state concentrations of H_2_ and H_2_O_2_ are visible (see Supporting Figure S3). The presence of the para-oxygen radical appears to substantially affect the testing parameters at extremely low dose rates up to 1 Gy/s (Supporting Figure S4). Excluding O^–^ together with HO_3_, O_3_, and O_3_^–^ does not cause additional changes (Supporting Figure S5). Interestingly, the exlusion of O appears to cause stronger alterations than removing O^–^, HO_3_, O_3_, and O_3_^–^ together (see Supporting Figure S6). Also, deactivating O appears to slightly violate the stability of the steady state concentrations of H_2_O_2_ and O_2_ for an initial pH of 3 up to 100 Gy/s. Hence, with regard to LP-TEM, both O and O^–^ are expected to be insignificant.

**Figure 2:**
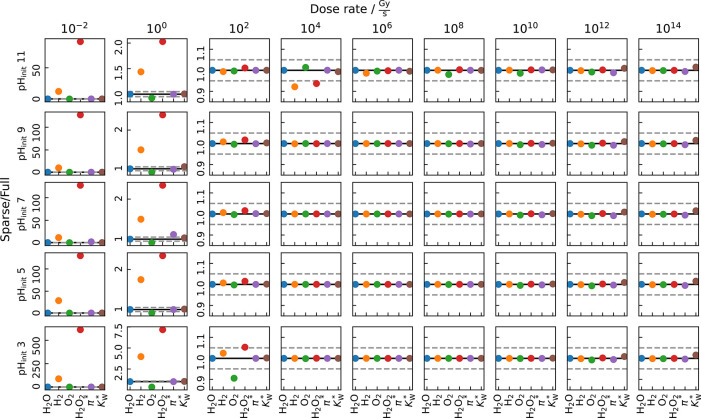
Relative deviation of the testing parameters of pure water sparsening down the kinetic model to 12 reactants (H_2_O, H^+^, OH^–^, O_2_, H, OH, H_2_O_2_, HO_2_, HO_2_^–^, O_2_^–^, and e_h_^–^). The solid line denotes a perfect agreement between the full and the sparsened model, whereas the dashed lines mark the 5% accuracy limit.

These findings also hold if these candidates are disabled simultaneously, yet with adding up the already noted parameter violations at low dose rates and initial pH far apart from 7, as demonstrated in Figure 2. This leaves a sparsened reaction set consisting of only 12 reactants (H_2_O, H^+^, OH^–^, H_2_, O_2_, H, OH, H_2_O_2_, HO_2_, HO_2_^–^, O_2_^–^, and e_h_^–^) enabling a reduction of the number of reactants by 29.4%. This translates to a 3.7-fold increase in computational speed (see Supporting Table S2 and Supporting Figure S20).

The set of residual species precisely matches the selected reactants in the temperature-dependent model for water radiolysis curated based on the work of Elliot and Bartels,^24^ justifying its usage for LP-TEM.

**Figure 3:**
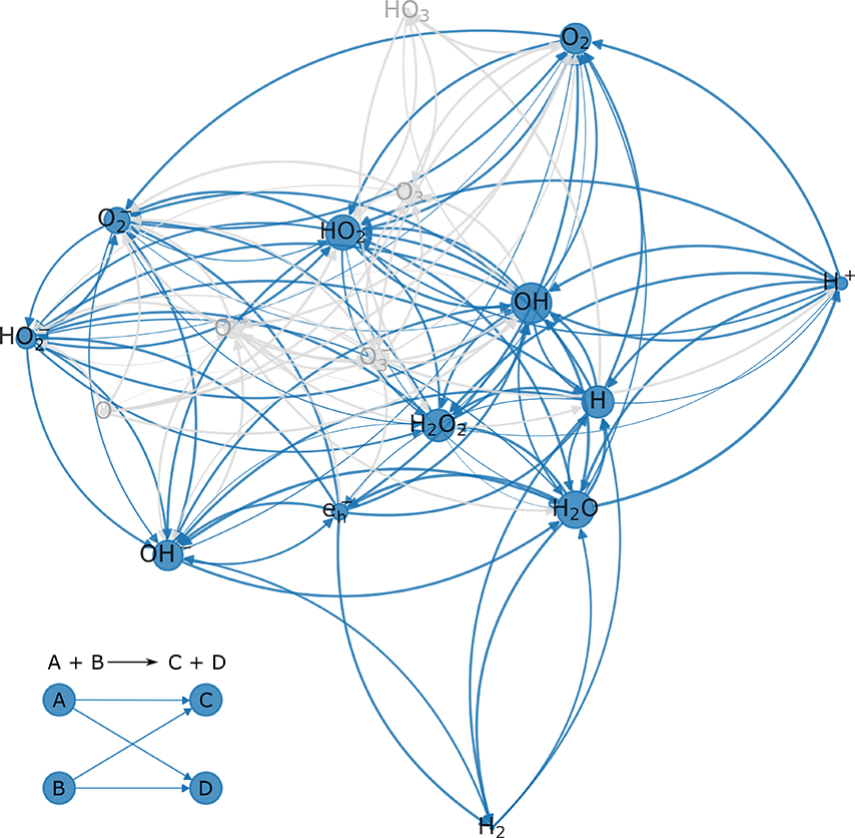
Graph representation of the sparsened reaction set for pure water. The gray nodes and edges illustrate deactivated reaction pathways.

Figure 3 provides a graph representation of this sparsened reaction set.^20,35^ The deactivated reactants and related reaction pathways are shaded in gray. The width of the edges is a measure of the logarithm of the rate constant of the respective reaction, while the size of the nodes is defined by their betweenness centrality (a measure of information flow through the network).^36^ It is visible that nodes with relatively small betweenness centralities are disabled if they are not guarded by the constraints defined above. However, it must be noted that this graph does not capture differences in initial concentrations, so that *a priori* all reactants are treated equally relevant. In consequence, we discuss below that estimating this relevance by graph analysis alone can be misleading if this assumption is invalid.

The power of radiation chemistry simulations becomes particularly prominent when applied systems are directly modeled. For the case of diluted, aqueous HAuCl_4_ solutions we were recently capable of demonstrating excellent agreement between simulations and experiments.^20^ To do so, we composed a kinetic model comprising 42 reactants. In the following, we will estimate the relevance of those by applying an approach similar to that elucidated above for pure water.

First, we extend our tracked main products to Cl^–^, Au^0^, and HCl. While Au^0^ (or abbreviated as Au) can be easily observed by precipitation^37^ and was simulated with quantitative agreement to our experimental observations,^20^ molecular HCl is expected to cause bubble-mediated etching of gold nanocrystals at solid–liquid–gas interfaces.^20,26^ Moreover, by assuming a strong acidic behavior of HAuCl_4_,^25^ AuCl_4_^–^ was treated as a species present prior to irradiation. This effectively caused all gold related species to become crucial in order to allow formation of Au^0^ by the pathway proposed by Dey et al.^38^

**Figure 4:**
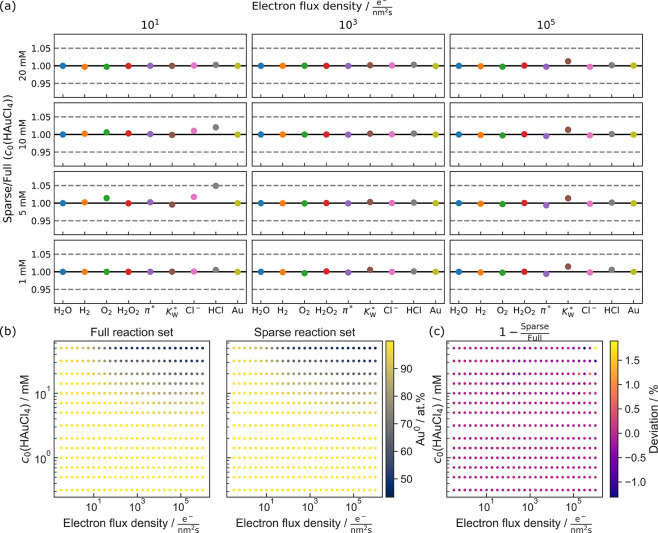
Sparsening of the HAuCl_4_ kinetic model for aqueous, diluted HAuCl_4_. The parameter space is adjusted to fit previous work.^20^ (a) Relative deviation of key parameters in the steady state for HAuCl_4_ simulations. The solid line denotes a perfect agreement between the full and the sparsened model, whereas the dashed lines mark the 5% accuracy limit. (b) Comparative steady state evolution of Au^0^ in aqueous HAuCl_4_ solution with the comprehensive and the sparse kinetic model. (c) Relative deviation between the data plotted in (b).

Second, we narrowed down the parameter space to the initial HAuCl_4_ concentrations and electron flux densities relevant to the LP-TEM-related studies evaluated in ref.^20^ The resulting range is shown in Figure 4a.

By using systematic spot checks (see Supporting Figures S7—S19), we could eliminate 20 of the 42 species without any drastic parameter failure (see Figure 4(a)), the number of reactants resembles a reduction by 47.6%. This translates to a 5.5-fold increase in computational speed (see again Supporting Table S2 and Supporting Figure S20).

Only at 10 e^–^/(nm^2^s) and 5 mM HAuCl_4_, we observed that HCl pushes the limits of its accuracy boundary. However, as this species is only a relevant product at high electron-flux densities and high initial HAuCl_4_ concentrations, we regard this as insignificant.

Figure 4b displays the relative amount of Au reduced to zero valency, on the one hand for the full parameter space simulated in our previous work (left),^20^ and on the other hand using the sparsened set (right). The two plots show a striking resemblance, which is supported by their relative deviations, as shown in Figure 4c. It shows that the accuracy remains within 2% for all data points. This means that all conclusions drawn from the comprehensive set are achievable with a substantially smaller set of ODEs.

**Figure 5:**
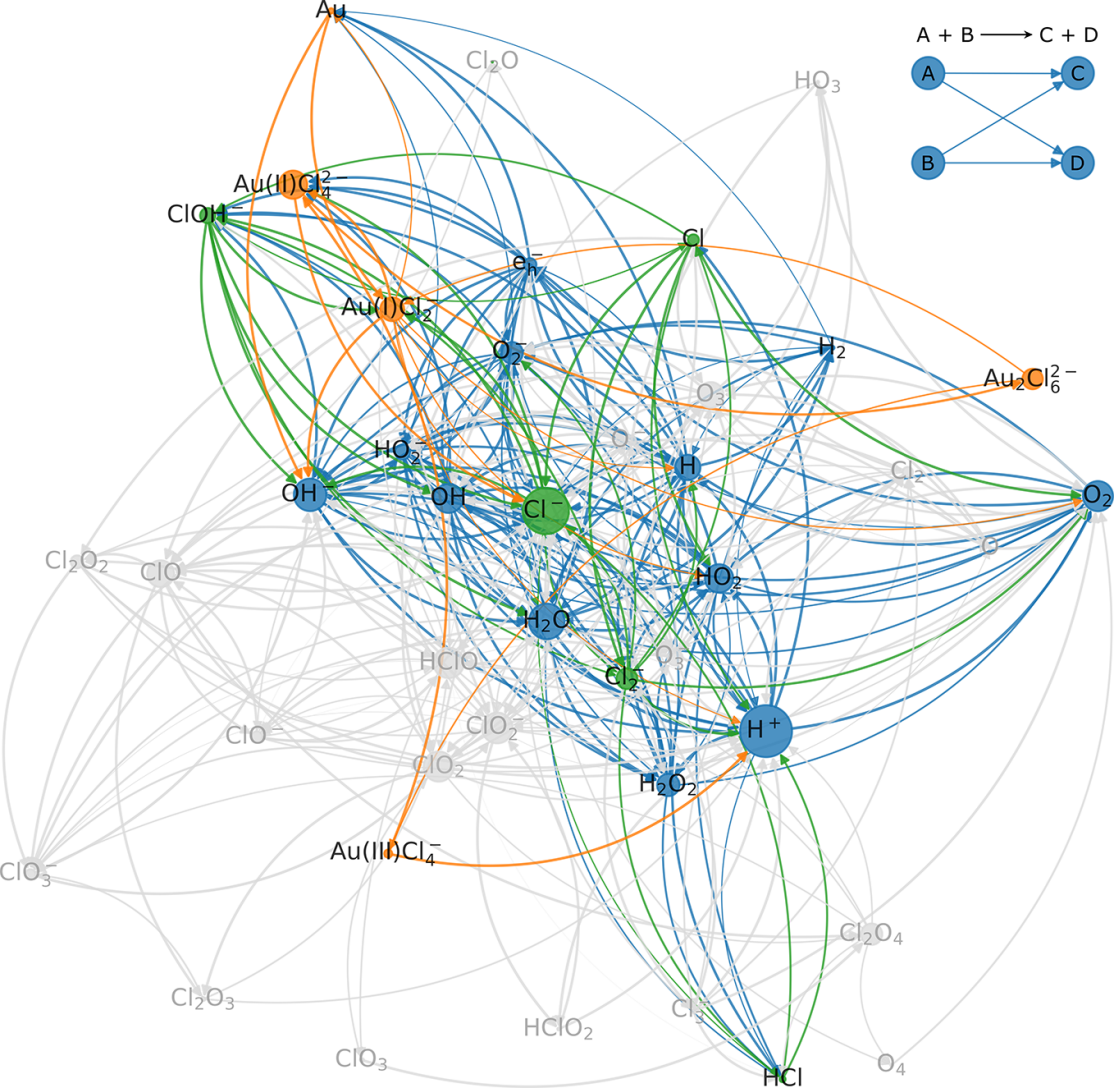
Graph representation of the aqueous HAuCl_4_ reaction set. The gray nodes and edges illustrate disabled pathways. Blue reactants denote chemicals consisting only of oxygen and/or hydrogen; orange ones contain Au, and green species label chlorine-containing reactants that do not include any gold atoms.

In particular, we were able to exclude Cl_2_, Cl_2_O, Cl_2_O_2_, Cl_2_O_3_, Cl_2_O_4_, Cl_3_^–^, ClO, ClO^–^, ClO_2_, ClO_2_^–^, ClO_3_, ClO_3_^–^, O_4_, HClO, and HClO_2_ on top of the species that were already deactivated for the water backbone discussed above. A corresponding graph is plotted in Figure 5. This leaves five purely chlorine-related reactants (green), namely, HCl, Cl^–^, Cl_2_^–^, and ClOH^–^, of which the first two species are bound by the set constraints.

Notably, the remaining three reactants do not stand out from the disabled ones in terms of betweenness centrality (node size). This could relate to the fact that most of them are multistep products during the irradiation of HAuCl_4_ solutions. Using different starting concentrations, e.g., by adding HClO to the reactor, would most likely alter the situation drastically.

On the other hand, the importance of ClOH^–^ is most likely related to the single oxidation pathway of Au available within the full reaction set (see reaction 186 of Supporting Table S1). Excluding ClOH^–^ would disable this reaction route and consequently heavily alter the obtained Au concentration (cf., Figure 4b, c). As a consequence, also Cl^–^, and HCl concentrations would be heavily affected (see Supporting Figure S19). Likewise, the Cl radical appears to substantially contribute to the formation of ClOH^–^ (see for instance reaction 164 of Supporting Table S1) which could potentially explain its relevance. Cl_2_^–^, in turn, not only is heavily entangled with Cl and ClOH^–^, but also provides prominent formation routes for HCl (see reactions 104, 111, and 138 of Supporting Table S1).

## 4 Discussion

Due to the high interconnectivity over multiple, connected equilibria, the precise reaction to determine the relevance of an individual reactant is not always feasible. While in some cases the importance is obvious due to the lack of alternative reaction pathways (such as in the case of gold oxidation by ClOH^–^), in many cases only speculations are possible that may help to understand the obtained results better. Yet, such speculations do not easily allow for predictions, as doing so is prone to drastically oversimplify the complexity within strongly interconnected kinetic models. To avoid erroneous conclusions which may distract further research, it is advised to rely on calculating the interplay of the whole reaction network simultaneously, as we do in this work. Yet, we note that big data analysis methods could provide an alternative possibility to interpret kinetic models, if solving the respective ODE system had to be avoided.^39^

Nonetheless, we still note a few general impressions. In order to exclude reactants without distorting the interplay within the network, it must be ensured that decay (and formation) pathways are maintained for all remaining species. This is particularly relevant for acid–base pairs. Also, the removal of secondary species could cause an artificial deactivation of only reductive or oxidative reaction pathways. This could suggest an artificial scavenging effect that may cause a false interpretation for experimental implications. Still, neglecting of secondary or tertiary products who are only formed by other low-concentration byproducts (e.g., Cl_2_O_3_ or Cl_2_O_4_) worked well as starting points in our case. On the other hand, chemicals that strongly react with primary species were less likely to be irrelevant to our metrics.

Our sparsened reaction set for pure water was able to serve as a reliable backbone for the more applied use case of diluted HAuCl_4_ solutions. This robustness suggests that it is applicable to more complex aqueous systems and could serve as a facile starting point for future kinetic simulations aiming at diluted aqueous systems under irradiation. Yet, there are a few limitations to account for.

As stated above, HCl pushed its accuracy limits under a parameter setting where it was only formed in spurs (see again Figure 4a). This case demonstrates that such extremely low-concentrated byproducts can undergo substantial changes in concentration due to the sparsening of the reaction set. If such an accuracy is required, we advise the usage of a comprehensive model or the inclusion of the respective species concentration as an additional constraint for sparsening.

**Figure 6:**
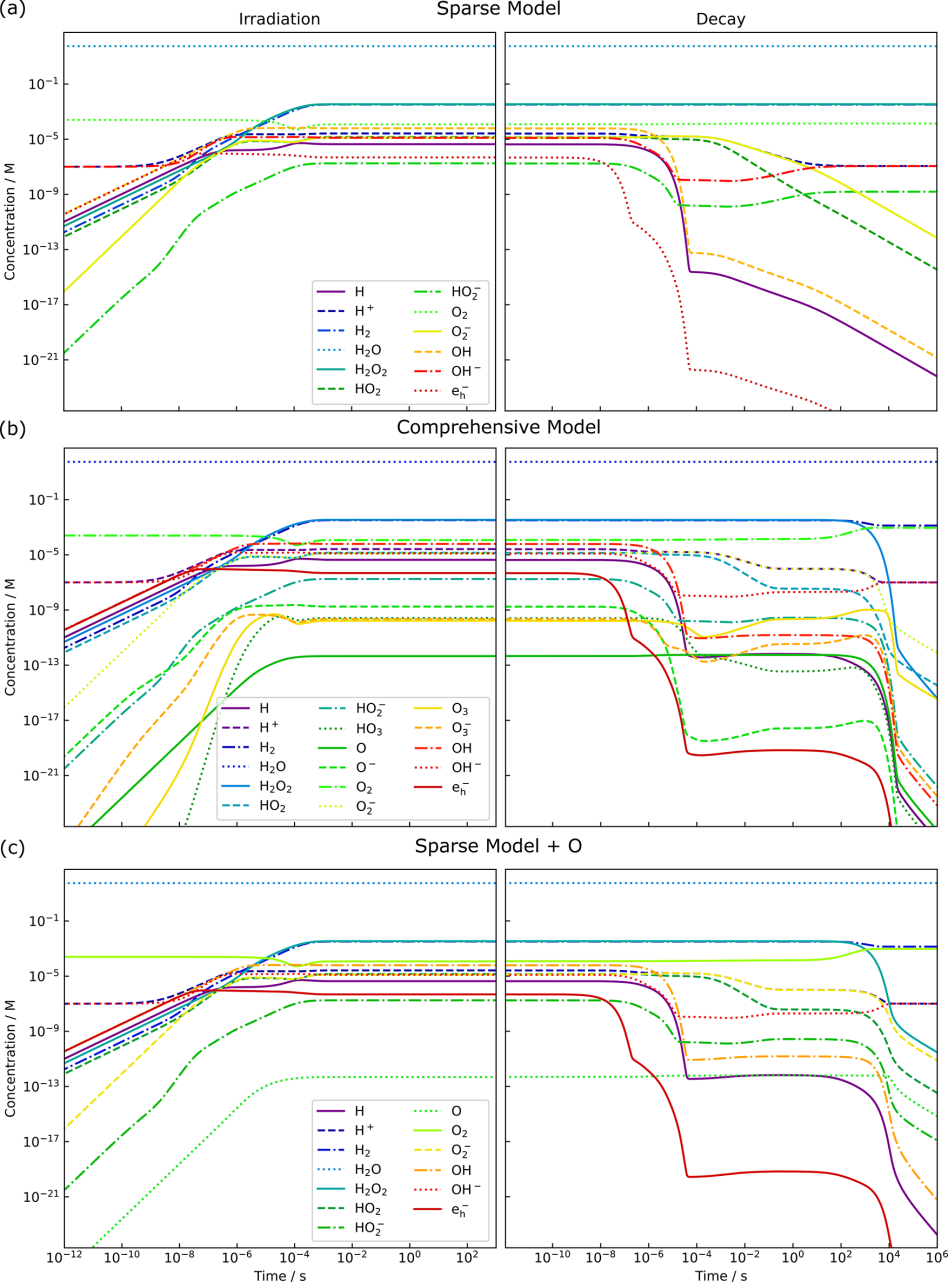
Evolution of concentrations for all reactants in pure water (left) under irradiation with an electron beam at a dose rate of 10^8^ Gy/s and subsequent concentration development without irradiation, i.e., due to beam blanking (steady state decay, right). The simulations were performed using (a) the sparsened model with 12 reactants and (b) the initial, comprehensive, and kinetic model. By incorporating O in the sparsened reaction set (c), the decay formation shown in (b) can be fairly reproduced (see Supporting Information for details).

As our chosen precision criteria all relate to a formed steady state under irradiation, effects regarding the decay of such a steady state may not be captured, in particular, outside of the irradiated volume. This is illustrated in Figure 6, which displays the formation of a steady state regime under irradiation (left) and its subsequent decay after switching off the electron beam (right) for the suggested sparse model with 12 reactants (a) and the initial, comprehensive reaction network (b). Electron-beam irradiation was simulated with a dose rate of 10^8^ Gy/s, a moderate magnitude during LP-TEM.

These long-term decay characteristics can differ substantially: While in the sparse reaction set H^+^ and OH^–^ settle after about 100 s, the initial model reveals a residual excitation of the pair until about 10^4^ s. The discrepancy becomes even more striking when regarding the main products (H_2_, H_2_O_2_, and O_2_) whose concentrations appear to remain at their excited steady state values when relying on the sparse reaction network. However, the comprehensive model reveals that they eventually start to decay after about 10^4^ s as well.

Notably, the widely used reaction set for characterization of radiation chemistry in LP-TEM of Schneider et al. neither describes such long-term decay characteristics.^10^ Although this limitation is directly stated within their work, it is additionally shown in Supporting Figure S21. Consequently, our sparse water set does not under-perform this important benchmark. Hence, it could be a feasible substitute for any modeling relying on the water set of Schneider et al.

Nonetheless, these features can be captured when reincorporating O into the sparsened reaction set (Figure 6c). Notably, this holds for a substantial fraction of the tested parameters for medium-to-high dose rates relevant to LP-TEM (see Supporting Information for details). However, this naturally decreases the speed achieved by sparsening. Yet, regarding O within the sparse reaction set causes an increased simulation duration by only 30% (see again Supporting Table S2 and Supporting Figure S20). Potential users should balance this longer duration with additional information depending on their requirements.

This is especially relevant when combining LP-TEM with complementary online analyses, such as coupled mass spectrometry to liquid flow set up, as suggested elsewhere,^40^ and as it is *lege artis* using other *in situ* methods for studying electrochemical processes.^41^ Here, care must be taken to adjust delay times and choice of radiation chemistry models accordingly if results outside of the irradiated area or after irradiation are of interest.

During our spot checks, dose rates were simulated up to values for which the concentration of the solvent begins to change notably, so that the assumption that the irradiation interacts only with H_2_O becomes questionable. This threshold is reported to be around 10^13^ Gy/s.^10,32^ Strictly speaking, such simulations must be regarded as a mere extrapolation of the parameter space, where the constraint still holds. However, as such dose rates (and even higher ones) are accessible in LP-TEM (i.e., during high-resolution LP-TEM investigations in graphene liquid cells), these extrapolations are an attempt toward providing useful information for such studies. Notably, none of the excluded reactants violated our precision limits exclusively under such conditions. Thus, this limitation does not affect the main conclusions that we draw from this work.

Furthermore, we emphasize that all of the presented results refer to steady-state concentrations within an isotropic, homogeneously irradiated volume. If such assumptions are not easily applicable, i.e., due to the requirement of describing diffusion of radiolysis products,^42^ the incorporation of nucleation sites^43^ or effects directly related to a scanning beam,^18,43^ different constraints could potentially be of interest. However, the workflow sketched here and the proposed sparse kinetic models could be used as building blocks for cost-effective heterogeneous simulations.

It is worth noting that albeit the G-values used within this work provided simulations with excellent agreement with experiments in LP-TEM,^20^ they appear not to fully explain phenomena during cryo-TEM^44^ or liquid-phase SEM.^34^ Yet, for sparsening the kinetic model, this is of minor importance, as shown in Supporting Figures S23 and S24. Consequently, the model with reduced complexity is believed to be applicable for adjacent high dose rate low LET irradiation,^47^ such as γ- or (hard) X-rays. Mind, however, that beyond low LET irradiation, more notably changed generation values apply that could alter starting conditions and therefore the interplay within the reaction network. Nonetheless, our work provides a workflow how to optimize such simulations with respect to accuracy and computational cost-effectiveness that can easily be applied to any kind of kinetic model.

In the end, we point out that predictive modeling of an unknown parameter space may still benefit from the usage of a comprehensive kinetic model. As soon as this is established, it can still be sparsened for cost-intensive follow-up studies, as demonstrated herein. However, during interpretation of experimental data, a sparse model can help to avoid gratuitously complicated conclusions.^45^ In this sense, ensuring simplicity by sparsening also serves as a promising tool for maintaining good scientific practice.

## 5 Conclusions

In summary, we present a systematic study to evaluate the relevance of individual reactants during solution radiation chemistry of LP-TEM. We show that for the buildup of steady-state concentrations of pure water, a reduced set of 12 species is sufficient, increasing the computational speed 3.7-fold. Subsequently, the approach is applied to simplify a comprehensive set on radiolytic gold formation by 48% while maintaining a precision of 98% and gaining a 5.5-fold increase in computation speed. However, long-term decay pathways are not necessarily captured by these sparsened models, justifying the usage of computationally more expensive models if such information is required. Our findings provide valuable insights for cost-effective radiation chemistry simulations, not only for LP-TEM but any system under irradiation.

## Supporting Information Available

Tabular representation of the kinetic models; additional spot checks for sparsening the pure water set; additional spot checks for sparsening the HAuCl_4_ set; runtime analysis; investigations on steady state decay; additional G-value test; additional references^46,47^ (PDF)
