# Supplementary material for: Precision of Radiation Chemistry Networks: Playing Jenga with Kinetic Models for Liquid-Phase Electron Microscopy
Source: Precis Chem. 2023 Dec 6;1(10):592–601. doi: 10.1021/prechem.3c00078 (PMC11503811; doi:10.1021/prechem.3c00078)
Supplement: Supplementary file 1 — pc3c00078_si_001.pdf [file pc3c00078_si_001.pdf]

Supporting Information

Precision of Radiation Chemistry Networks: Playing Jenga with Kinetic Models for Liquid-Phase Electron Microscopy

Birk Fritsch*[,a], Paolo Malgaretti[a], Jens Harting[a,b], Karl J. J. Mayrhofer[a], Andreas Hutzler*[,a]

*corresponding authors. Birk Fritsch: b.fritsch@fz-juelich.de, Andreas Hutzler: a.hutzler@fz-juelich.de

Affiliations:

[a] Forschungszentrum Jülich GmbH, Helmholtz Institute Erlangen-Nürnberg for Renewable Energy (IEK-11), Cauerstr. 1, 91058 Erlangen, Germany

[b] Department of Chemical and Biological Engineering and Department of Physics, Friedrich-Alexander-Universität Erlangen-Nürnberg, Cauerstr. 1, 91058 Erlangen, Germany

Kinetic Model

Table S1: Kinetic model for radiation chemistry of pure water (reaction 1 – 86) and aqueous $HAuCl_4$ solutions (all reactions). Note, that k is the respective kinetic constant in unit of mol^{-n+1} $dm^{3(n-1)}$ s^{-1} with n as reaction order. The sparsened version is labelled by the rightest column, where "✓" denotes that the reaction is part of the sparsened reaction set. "X" labels excluded reactions. Moreover, "(D)" denotes that the reaction was regarded for the decay estimation elucidated below.

	Reaction			***k***	Source	Sparse version
**1**	H_2O	→	$H^+ + OH^-$	$2.599 \cdot 10^{-5}$	[1]	✓
**2**	$H^+ + OH^-$	→	H_2O	$1.43 \cdot 10^{11}$	[1]	✓
**3**	H_2O_2	→	$H^+ + HO_2^-$	$1.119 \cdot 10^{-1}$	[1]	✓
**4**	$H^+ + HO_2^-$	→	H_2O_2	$5 \cdot 10^{10}$	[1]	✓
**5**	$H_2O_2 + OH^-$	→	$HO_2^- + H_2O$	$1.3 \cdot 10^{10}$	[1]	✓
**6**	$HO_2^- + H_2O$	→	$H_2O_2 + OH^-$	$5.82 \cdot 10^{7}$	[1]	✓
**7**	$e_h^- + H_2O$	→	$H + OH^-$	$1.9 \cdot 10^{1}$	[1]	✓
**8**	$H + OH^-$	→	$e_h^- + H_2O$	$2.2 \cdot 10^{7}$	[1]	✓
**9**	H	→	$e_h^- + H^+$	$3.9 \cdot 10^{0}$	[1]	✓
**10**	$e_h^- + H^+$	→	H	$2.3 \cdot 10^{10}$	[1]	✓
**11**	$OH + OH^-$	→	$O^- + H_2O$	$1.3 \cdot 10^{10}$	[1]	X
**12**	$O^- + H_2O$	→	$OH + OH^-$	$1 \cdot 10^{8}$	[1]	X
**13**	OH	→	$O^- + H^+$	$1.259 \cdot 10^{-1}$	[1]	X
**14**	$O^- + H^+$	→	OH	$1 \cdot 10^{11}$	[1]	X
**15**	HO_2	→	$O_2^- + H^+$	$1.346 \cdot 10^{6}$	[1]	✓

	Reaction			k	Source	Sparse version
16	$O_2^- + H^+$	→	HO_2	$5 \cdot 10^{10}$	[1]	✓
17	$HO_2 + OH^-$	→	$O_2^- + H_2O$	$5 \cdot 10^{10}$	[1]	✓
18	$O_2^- + H_2O$	→	$HO_2 + OH^-$	$1.862 \cdot 10^{1}$	[1]	✓
19	$e_h^- + OH$	→	OH^-	$3 \cdot 10^{10}$	[1]	✓
20	$e_h^- + H_2O_2$	→	$OH + OH^-$	$1.1 \cdot 10^{10}$	[1]	✓
21	$e_h^- + O_2^- + H_2O$	→	$HO_2^- + OH^-$	$1.3 \cdot 10^{10}$	[1]	✓
22	$e_h^- + HO_2$	→	HO_2^-	$2 \cdot 10^{10}$	[1]	✓
23	$e_h^- + O_2$	→	O_2^-	$1.9 \cdot 10^{10}$	[1]	✓
24	$2\ e_h^- + 2\ H_2O$	→	$H_2 + 2\ OH^-$	$5.5 \cdot 10^{9}$	[1]	✓
25	$e_h^- + H + H_2O$	→	$H_2 + OH^-$	$2.5 \cdot 10^{10}$	[1]	✓
26	$e_h^- + HO_2^-$	→	$O^- + OH^-$	$3.5 \cdot 10^{9}$	[1]	X
27	$e_h^- + O^- + H_2O$	→	$OH^- + OH^-$	$2.2 \cdot 10^{10}$	[1]	X
28	$e_h^- + O_3^- + H_2O$	→	$O_2 + OH^- + OH^-$	$1.6 \cdot 10^{10}$	[1]	X
29	$e_h^- + O_3$	→	O_3^-	$3.6 \cdot 10^{10}$	[1]	X
30	$H + H_2O$	→	$H_2 + OH$	$1.1 \cdot 10^{1}$	[1]	✓
31	$H + O^-$	→	OH^-	$1 \cdot 10^{10}$	[1]	X
32	$H + HO_2^-$	→	$OH + OH^-$	$9 \cdot 10^{7}$	[1]	✓
33	$H + O_3^-$	→	$OH^- + O_2$	$1 \cdot 10^{10}$	[1]	X
34	$2\ H$	→	H_2	$7.8 \cdot 10^{9}$	[1]	✓
35	$H + OH$	→	H_2O	$7 \cdot 10^{9}$	[1]	✓
36	$H + H_2O_2$	→	$OH + H_2O$	$9 \cdot 10^{7}$	[1]	✓
37	$H + O_2$	→	HO_2	$2.1 \cdot 10^{10}$	[1]	✓
38	$H + HO_2$	→	H_2O_2	$1.8 \cdot 10^{10}$	[1]	✓
39	$H + O_2^-$	→	HO_2^-	$1.8 \cdot 10^{10}$	[1]	✓
40	$H + O_3$	→	HO_3	$3.8 \cdot 10^{10}$	[1]	X
41	$2\ OH$	→	H_2O_2	$3.6 \cdot 10^{9}$	[1]	✓
42	$OH + HO_2$	→	$H_2O + O_2$	$6 \cdot 10^{9}$	[1]	✓
43	$OH + O_2^-$	→	$OH^- + O_2$	$8.2 \cdot 10^{9}$	[1]	✓
44	$OH + H_2$	→	$H + H_2O$	$4.3 \cdot 10^{7}$	[1]	✓
45	$OH + H_2O_2$	→	$HO_2 + H_2O$	$2.7 \cdot 10^{7}$	[1]	✓
46	$OH + O^-$	→	HO_2^-	$2.5 \cdot 10^{10}$	[1]	X
47	$OH + HO_2^-$	→	$HO_2 + OH^-$	$7.5 \cdot 10^{9}$	[1]	✓
48	$OH + O_3^-$	→	$O_3 + OH^-$	$2.6 \cdot 10^{9}$	[1]	X
49	$OH + O_3^-$	→	$2\ O_2^- + H^+$	$6 \cdot 10^{9}$	[1]	X
50	$OH + O_3$	→	$HO_2 + O_2$	$1.1 \cdot 10^{8}$	[1]	X
51	$HO_2 + O_2^-$	→	$HO_2^- + O_2$	$8 \cdot 10^{7}$	[1]	✓

	Reaction			k	Source	Sparse version
52	$HO_2 + HO_2$	→	$H_2O_2 + O_2$	$7 \cdot 10^{5}$	1	✓
53	$HO_2 + O^-$	→	$O_2 + OH^-$	$6 \cdot 10^{9}$	1	X
54	$HO_2 + H_2O_2$	→	$OH + O_2 + H_2O$	$5 \cdot 10^{-1}$	1	✓
55	$HO_2 + HO_2^-$	→	$OH + O_2 + OH^-$	$5 \cdot 10^{-1}$	1	✓
56	$HO_2 + O_3^-$	→	$O_2 + O_2 + OH^-$	$6 \cdot 10^{9}$	1	X
57	$HO_2 + O_3$	→	$HO_3 + O_2$	$5 \cdot 10^{8}$	1	X
58	$2\ O_2^- + 2\ H_2O$	→	$H_2O_2 + O_2 + 2\ OH^-$	$1 \cdot 10^{2}$	1	✓
59	$O_2^- + O^- + H_2O$	→	$O_2 + 2\ OH^-$	$6 \cdot 10^{8}$	1	X
60	$O_2^- + H_2O_2$	→	$OH + O_2 + OH^-$	$1.3 \cdot 10^{-1}$	1	✓
61	$O_2^- + HO_2^-$	→	$O^- + O_2 + OH^-$	$1.3 \cdot 10^{-1}$	1	X
62	$O_2^- + O_3^- + H_2O$	→	$O_2 + O_2 + 2\ OH^-$	$1 \cdot 10^{4}$	1	X
63	$O_2^- + O_3$	→	$O_3^- + O_2$	$1.5 \cdot 10^{9}$	1	X
64	$2\ O^- + H_2O$	→	$HO_2^- + OH^-$	$1 \cdot 10^{9}$	1	X
65	$O^- + O_2$	→	O_3^-	$3.6 \cdot 10^{9}$	1	X
66	$O^- + H_2$	→	$H + OH^-$	$8 \cdot 10^{7}$	1	X
67	$O^- + H_2O_2$	→	$O_2^- + H_2O$	$5 \cdot 10^{8}$	1	X
68	$O^- + HO_2^-$	→	$O_2^- + OH^-$	$4 \cdot 10^{8}$	1	X
69	$O^- + O_3^-$	→	$O_2^- + O_2^-$	$7 \cdot 10^{8}$	1	X
70	$O^- + O_3$	→	$O_2^- + O_2$	$5 \cdot 10^{9}$	1	X
71	O_3^-	→	$O_2 + O^-$	$3.3 \cdot 10^{3}$	1	X
72	$O_3^- + H^+$	→	$O_2 + OH$	$9 \cdot 10^{10}$	1	X
73	HO_3	→	$O_2 + OH$	$1.1 \cdot 10^{5}$	1	X
74	H_2O_2	→	$H_2O + O$	$1 \cdot 10^{-3}$	1	X
75	$2\ O$	→	O_2	$2.2 \cdot 10^{10}$	2	X, (D)
76	O_3	→	$O_2 + O$	$3 \cdot 10^{-6}$	1	X
77	$2\ O_3^- + H_2O$	→	$OH^- + HO_2^- + 2\ O_2$	$1 \cdot 10^{4}$	1	X
78	$2\ HO_3$	→	$H_2O_2 + 2\ O_2$	$5 \cdot 10^{9}$	1	X
79	$O_3 + OH^-$	→	$HO_2^- + O_2$	$1 \cdot 10^{2}$	1	X
80	$O_2 + O$	→	O_3	$4 \cdot 10^{9}$	1	X
81	$H_2O_2 + O$	→	$OH + HO_2$	$1.6 \cdot 10^{9}$	1	X, (D)
82	$O + HO_2^-$	→	$OH + O_2^-$	$5.3 \cdot 10^{9}$	1	X, (D)
83	$O + OH^-$	→	HO_2^-	$4.2 \cdot 10^{8}$	1	X, (D)
84	$O + OH$	→	HO_2	$2 \cdot 10^{10}$	2	X, (D)
85	$O + HO_2$	→	$OH + O_2$	$2 \cdot 10^{10}$	2	X, (D)
86	$O + H$	→	OH	$2 \cdot 10^{10}$	2	X, (D)
87	$OH + Cl^-$	→	$ClOH^-$	$4.3 \cdot 10^{9}$	1	✓

	Reaction			k	Source	Sparse version
**88**	$OH + HClO$	→	$ClO + H_2O$	$9 \cdot 10^{9}$	1	X
**89**	$OH + ClO_2^- + H^+$	→	$ClO_2 + H_2O$	$6.3 \cdot 10^{9}$	1	X
**90**	$e_h^- + Cl$	→	Cl^-	$1 \cdot 10^{10}$	1	✓
**91**	$e_h^- + Cl_2^-$	→	$2\ Cl^-$	$1 \cdot 10^{10}$	1	✓
**92**	$e_h^- + ClOH^-$	→	$Cl^- + OH^-$	$1 \cdot 10^{10}$	1	✓
**93**	$e_h^- + HClO$	→	$ClOH^-$	$5.3 \cdot 10^{10}$	1	X
**94**	$e_h^- + Cl_2$	→	Cl_2^-	$1 \cdot 10^{10}$	1	X
**95**	$e_h^- + Cl_3^-$	→	$Cl_2^- + Cl^-$	$1 \cdot 10^{10}$	1	X
**96**	$e_h^- + ClO_2^- + H^+$	→	$ClO + OH^-$	$4.5 \cdot 10^{10}$	1	X
**97**	$e_h^- + ClO_3^- + H^+$	→	$ClO_2 + OH^-$	$1 \cdot 10^{10}$	1	X
**98**	$H + Cl$	→	$Cl^- + H^+$	$1 \cdot 10^{10}$	1	✓
**99**	$H + Cl_2^-$	→	$2\ Cl^- + H^+$	$8 \cdot 10^{9}$	1	✓
**100**	$H + ClOH^-$	→	$Cl^- + H_2O$	$1 \cdot 10^{10}$	1	✓
**101**	$H + Cl_2$	→	$Cl_2^- + H^+$	$7 \cdot 10^{9}$	1	X
**102**	$H + HClO$	→	$ClOH^- + H^+$	$1 \cdot 10^{10}$	1	X
**103**	$H + Cl_3^-$	→	$Cl_2^- + Cl^- + H^+$	$1 \cdot 10^{10}$	1	X
**104**	$HO_2 + Cl_2^-$	→	$Cl^- + HCl + O_2$	$4 \cdot 10^{9}$	1	✓
**105**	HCl	→	$Cl^- + H^+$	$5 \cdot 10^{5}$	1	✓
**106**	$Cl^- + H^+$	→	HCl	$6.29 \cdot 10^{-1}$	1	✓
**107**	$HO_2 + Cl_2$	→	$Cl_2^- + O_2 + H^+$	$1 \cdot 10^{9}$	1	X
**108**	$HO_2 + Cl_3^-$	→	$Cl_2^- + HCl + O_2$	$1 \cdot 10^{9}$	1	X
**109**	$O_2^- + Cl_2^-$	→	$2\ Cl^- + O_2$	$1.2 \cdot 10^{10}$	1	✓
**110**	$O_2^- + HClO$	→	$ClOH^- + O_2$	$7.5 \cdot 10^{6}$	1	X
**111**	$H_2O_2 + Cl_2^-$	→	$2\ HCl + O_2^-$	$1.4 \cdot 10^{5}$	1	✓
**112**	$H_2O_2 + Cl_2$	→	$HO_2 + Cl_2^- + H^+$	$1.9 \cdot 10^{2}$	1	X
**113**	$H_2O_2 + HClO$	→	$HCl + H_2O + O_2$	$1.7 \cdot 10^{5}$	1	X
**114**	$OH^- + Cl_2^-$	→	$ClOH^- + Cl^-$	$7.3 \cdot 10^{6}$	1	✓
**115**	$OH^- + Cl_2$	→	$HClO + Cl^-$	$6 \cdot 10^{8}$	1	X
**116**	$H^+ + ClOH^-$	→	$Cl + H_2O$	$2.1 \cdot 10^{10}$	1	✓
**117**	$H_2O + Cl_2O_2$	→	$HClO + ClO_2^- + H^+$	$1 \cdot 10^{4}$	1	X
**118**	$H_2O + Cl_2O$	→	$2\ HClO$	$1 \cdot 10^{2}$	1	X
**119**	$H_2O + Cl_2O_4$	→	$ClO_2^- + ClO_3^- + 2\ H^+$	$1 \cdot 10^{2}$	1	X
**120**	$H_2O + Cl_2O_4$	→	$HClO + HCl + O_4$	$1 \cdot 10^{2}$	1	X
**121**	O_4	→	$2\ O_2$	$1 \cdot 10^{5}$	1	X
**122**	$Cl^- + Cl$	→	Cl_2^-	$2.1 \cdot 10^{10}$	1	✓
**123**	$Cl^- + ClOH^-$	→	$Cl_2^- + OH^-$	$9 \cdot 10^{4}$	1	✓

	Reaction			k	Source	Sparse version
124	$Cl^- + HClO$	→	$Cl_2 + OH^-$	$1 \cdot 10^{1}$	[1]	X
125	$Cl^- + Cl_2$	→	Cl_3^-	$1 \cdot 10^{4}$	[1]	X
126	$ClOH^-$	→	$OH + Cl^-$	$6.1 \cdot 10^{9}$	[1]	✓
127	Cl_2^-	→	$Cl + Cl^-$	$1.1 \cdot 10^{5}$	[1]	✓
128	$2\ Cl_2^-$	→	$Cl_3^- + Cl^-$	$7 \cdot 10^{9}$	[1]	X
129	Cl_3^-	→	$Cl_2 + Cl^-$	$5 \cdot 10^{4}$	[1]	X
130	$2\ ClO$	→	Cl_2O_2	$1.5 \cdot 10^{10}$	[1]	X
131	$2\ ClO_2$	→	Cl_2O_4	$1 \cdot 10^{2}$	[1]	X
132	$Cl_2O_2 + ClO_2^-$	→	$ClO_3^- + Cl_2O$	$1 \cdot 10^{2}$	[1]	X
133	$2\ HClO$	→	$Cl^- + ClO_2^- + 2\ H^+$	$6 \cdot 10^{-9}$	[1]	X
134	$ClO_2^- + HClO$	→	$Cl^- + ClO_3^- + H^+$	$9 \cdot 10^{-7}$	[1]	X
135	$2\ HClO$	→	$O_2 + 2\ HCl$	$3 \cdot 10^{-10}$	[1]	X
136	$HClO + Cl^- + H^+$	→	$Cl_2 + H_2O$	$9 \cdot 10^{3}$	[1]	X
137	$Cl_2 + H_2O$	→	$HClO + Cl^- + H^+$	$1.5 \cdot 10^{1}$	[1]	X
138	$Cl_2^- + H_2$	→	$H + HCl + Cl^-$	$4.3 \cdot 10^{5}$	[1]	✓
139	$2\ Cl$	→	Cl_2	$8.8 \cdot 10^{7}$	[1]	X
140	$ClO_2 + O_3$	→	$O_2 + ClO_3$	$1.1 \cdot 10^{3}$	[1]	X
141	$ClO_2 + OH$	→	$ClO_3^- + H^+$	$4 \cdot 10^{9}$	[1]	X
142	$ClO_2 + O^-$	→	ClO_3^-	$2.7 \cdot 10^{9}$	[1]	X
143	$ClO_2 + O_3^-$	→	$O_2 + ClO_3^-$	$1.8 \cdot 10^{5}$	[1]	X
144	$ClO_2 + O_3^-$	→	$O_3 + ClO_2^-$	$1.8 \cdot 10^{5}$	[1]	X
145	$ClO_2^- + O_3$	→	$O_3^- + ClO_2$	$4 \cdot 10^{6}$	[1]	X
146	ClO_2	→	$O_2 + Cl$	$6.7 \cdot 10^{9}$	[1]	X
147	$HClO$	→	$H^+ + ClO^-$	$2 \cdot 10^{3}$	[1]	X
148	$H^+ + ClO^-$	→	$HClO$	$5 \cdot 10^{10}$	[1]	X
149	$HClO_2$	→	$H^+ + ClO_2^-$	$9.53 \cdot 10^{8}$	[1]	X
150	$H^+ + ClO_2^-$	→	$HClO_2$	$5 \cdot 10^{10}$	[1]	X
151	$Cl + O_3^-$	→	$Cl^- + O_3$	$1 \cdot 10^{9}$	[1]	X
152	$ClO + O_3^-$	→	$ClO^- + O_3$	$1 \cdot 10^{9}$	[1]	X
153	$Cl_2^- + ClO_2$	→	$Cl_2O_2 + Cl^-$	$1 \cdot 10^{9}$	[1]	X
154	$Cl + ClO_2$	→	Cl_2O_2	$1 \cdot 10^{9}$	[1]	X
155	$ClO + ClO_2^-$	→	$ClO^- + ClO_2$	$9.4 \cdot 10^{8}$	[1]	X
156	$ClO^- + O^- + H^+$	→	$ClO + OH^-$	$2.3 \cdot 10^{8}$	[1]	X
157	$Cl^- + H_2O_2$	→	$ClO^- + H_2O$	$1.8 \cdot 10^{-9}$	[1]	X
158	$Cl^- + H_2O_2 + H^+$	→	$HClO + H_2O$	$8.3 \cdot 10^{-7}$	[1]	X
159	$ClO^- + H_2O_2$	→	$Cl^- + O_2 + H_2O$	$3.4 \cdot 10^{3}$	[1]	X

	Reaction			k	Source	Sparse version
**160**	$HClO + HO_2^-$	→	$Cl^- + O_2 + H_2O$	$4.4 \cdot 10^7$	[1]	✗
**161**	$Cl_2 + HO_2^-$	→	$2\ Cl^- + O_2 + H^+$	$1.1 \cdot 10^8$	[1]	✗
**162**	$Cl + H_2O_2$	→	$Cl^- + H^+ + HO_2$	$2 \cdot 10^9$	[1]	✓
**163**	$Cl + HO_2$	→	$Cl^- + H^+ + O_2$	$3.1 \cdot 10^9$	[1]	✓
**164**	$Cl + OH^-$	→	$ClOH^-$	$1.8 \cdot 10^{10}$	[1]	✓
**165**	$ClO_2 + H_2O_2$	→	$ClO_2^- + H^+ + HO_2$	$4 \cdot 10^0$	[1]	✗
**166**	$ClO_2 + HO_2^-$	→	$ClO_2^- + HO_2$	$1.3 \cdot 10^5$	[1]	✗
**167**	$ClO_2 + HO_2$	→	$ClO_2^- + H^+ + O_2$	$1 \cdot 10^6$	[1]	✗
**168**	$ClO_2 + O_2^-$	→	$ClO_2^- + O_2$	$3 \cdot 10^9$	[1]	✗
**169**	$ClO_2^- + O_2^-$	→	$ClO^- + O^- + O_2$	$4 \cdot 10^1$	[1]	✗
**170**	$ClO + ClO_2$	→	Cl_2O_3	$7.4 \cdot 10^9$	[1]	✗
**171**	$ClO + ClO_3$	→	Cl_2O_4	$7.4 \cdot 10^9$	[1]	✗
**172**	$Cl_2O_2 + OH^-$	→	$Cl^- + ClO_3^- + H^+$	$1 \cdot 10^{10}$	[1]	✗
**173**	$Cl_2O_3 + H_2O$	→	$HClO + ClO_3^- + H^+$	$1 \cdot 10^4$	[1]	✗
**174**	$ClOH^-$	→	$Cl + OH^-$	$2.3 \cdot 10^1$	[1]	✓
**175**	$Cl + H_2O$	→	$ClOH^- + H^+$	$1.8 \cdot 10^5$	[1]	✓
**176**	$Cl_2^- + O_3$	→	$ClO + Cl^- + O_2$	$9 \cdot 10^7$	[1]	✗
**177**	$Au(I)Cl_2^- + 2\ Cl^- + OH$	→	$Au(II)Cl_4^{2-} + OH^-$	$2.6 \cdot 10^9$	[1]	✓
**178**	$Au(III)Cl_4^- + H$	→	$Au(II)Cl_4^{2-} + H^+$	$5.7 \cdot 10^9$	[1]	✓
**179**	$Au(III)Cl_4^- + e_h^-$	→	$Au(II)Cl_4^{2-}$	$5.7 \cdot 10^9$	[1]	✓
**180**	$2\ Au(II)Cl_4^{2-}$	→	$Au_2Cl_6^{2-} + 2\ Cl^-$	$1.45 \cdot 10^8$	[1]	✓
**181**	$Au(I)Cl_2^- + e_h^-$	→	$Au + 2\ Cl^-$	$8 \cdot 10^9$	[1]	✓
**182**	$Au(I)Cl_2^- + H$	→	$Au + H^+ + 2\ Cl^-$	$8 \cdot 10^9$	[1]	✓
**183**	$Au(I)Cl_2^- + HO_2^-$	→	$Au + HO_2 + 2\ Cl^-$	$1.89 \cdot 10^0$	[1]	✓
**184**	$Au(I)Cl_2^- + H_2$	→	$Au + H^+ + H + 2\ Cl^-$	$7.4 \cdot 10^{-3}$	[1]	✓
**185**	$Au(I)Cl_2^- + O_2^-$	→	$Au + O_2 + 2\ Cl^-$	$1.89 \cdot 10^0$	[1]	✓
**186**	$Au + ClOH^- + Cl^-$	→	$Au(I)Cl_2^- + OH^-$	$1.83 \cdot 10^9$	[1]	✓
**187**	$Au_2Cl_6^{2-}$	→	$Au(I)Cl_2^- + Au(III)Cl_4^-$	$1 \cdot 10^2$	[1]	✓

Additional spot checks for sparsening the pure water set

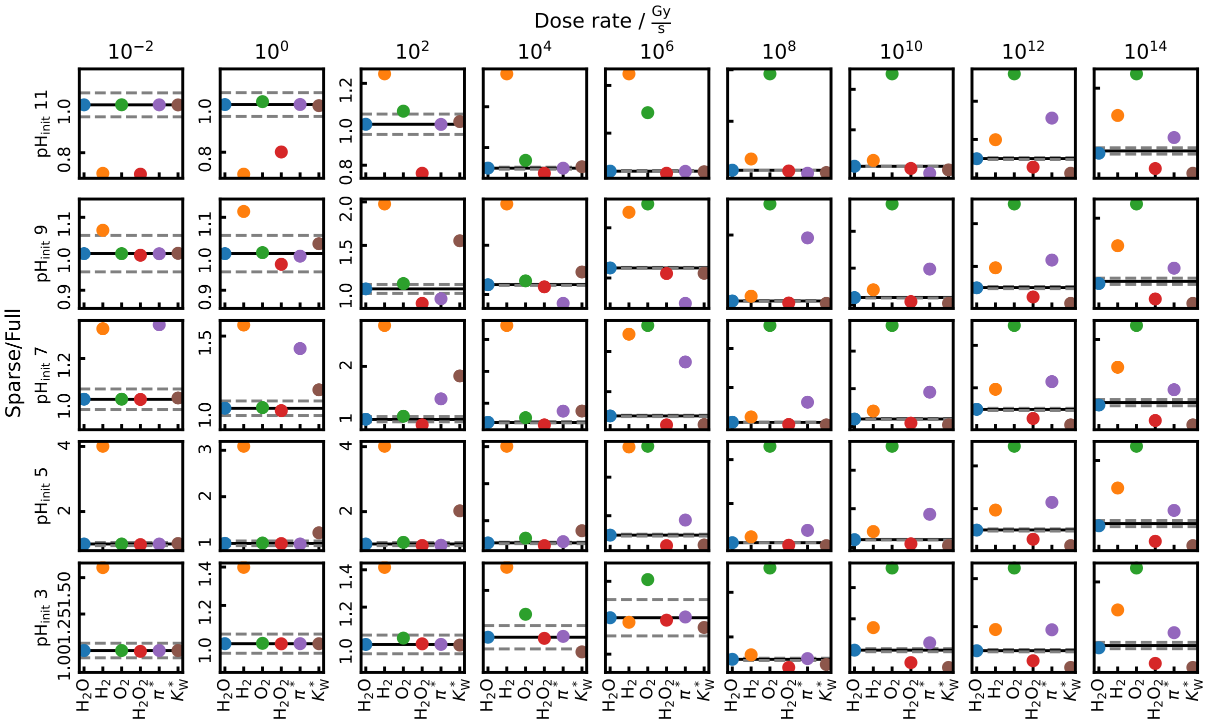

Figure S1: Excluded HO_2^-.

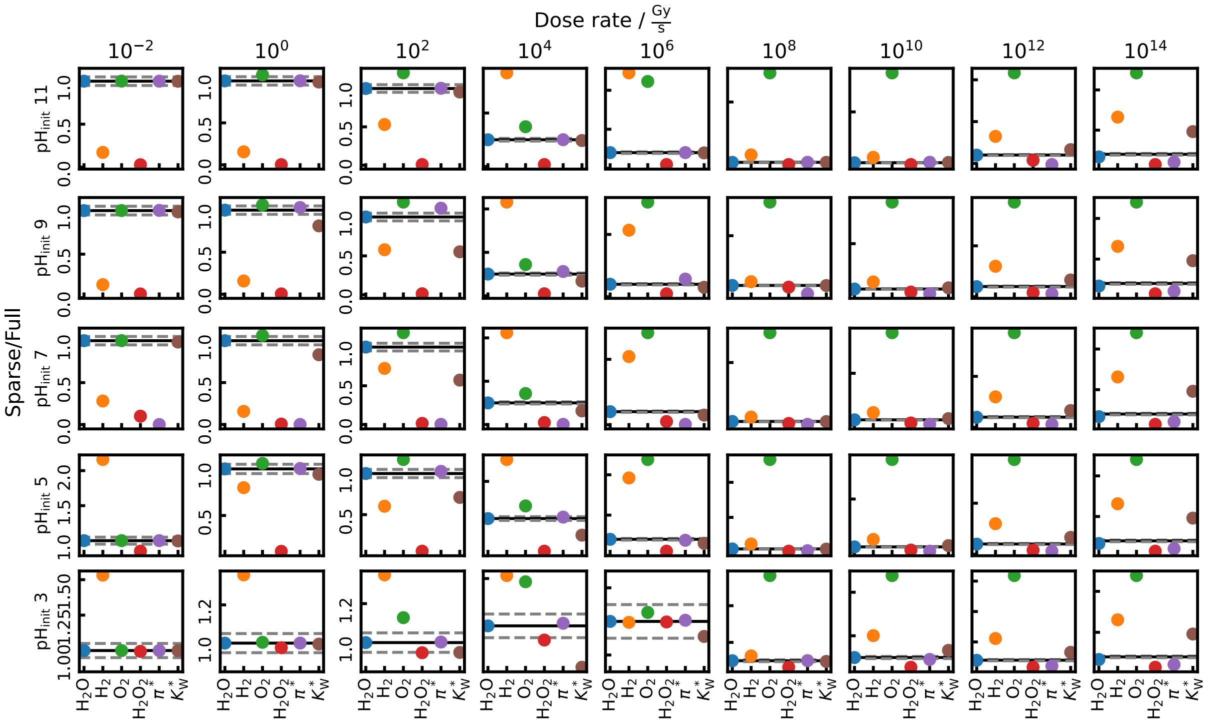

Figure S2: Excluded O_2^-.

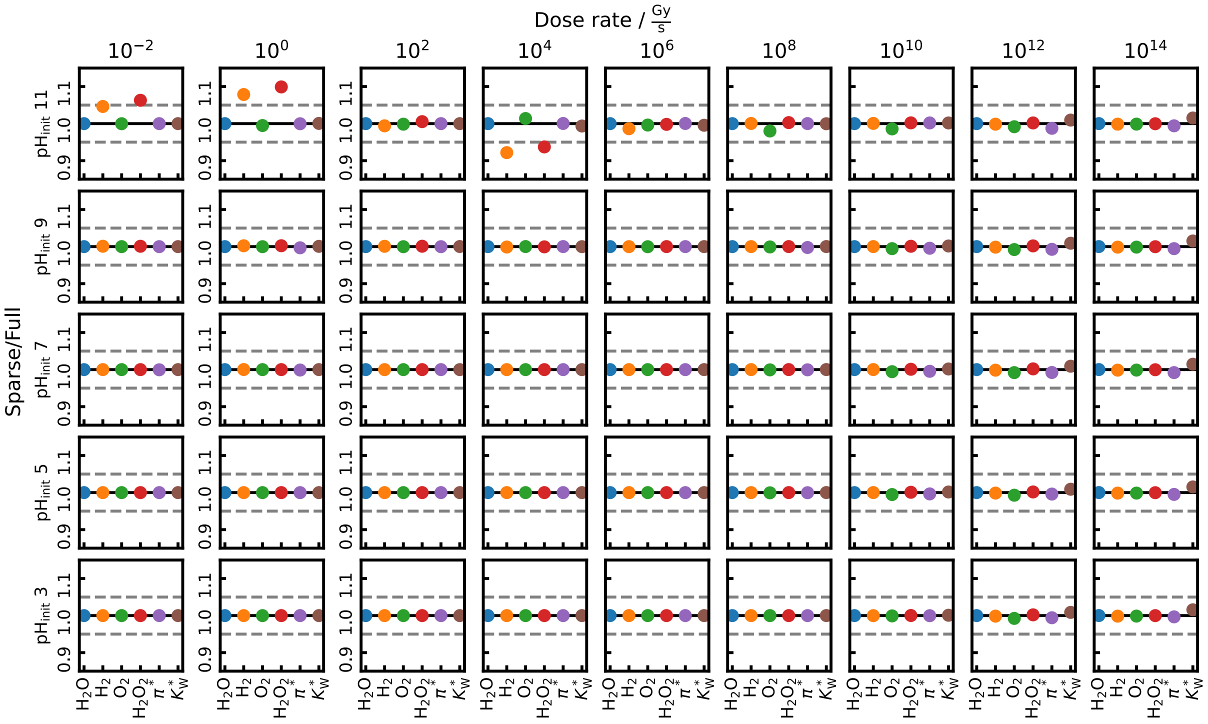

Figure S3: Excluded O^-.

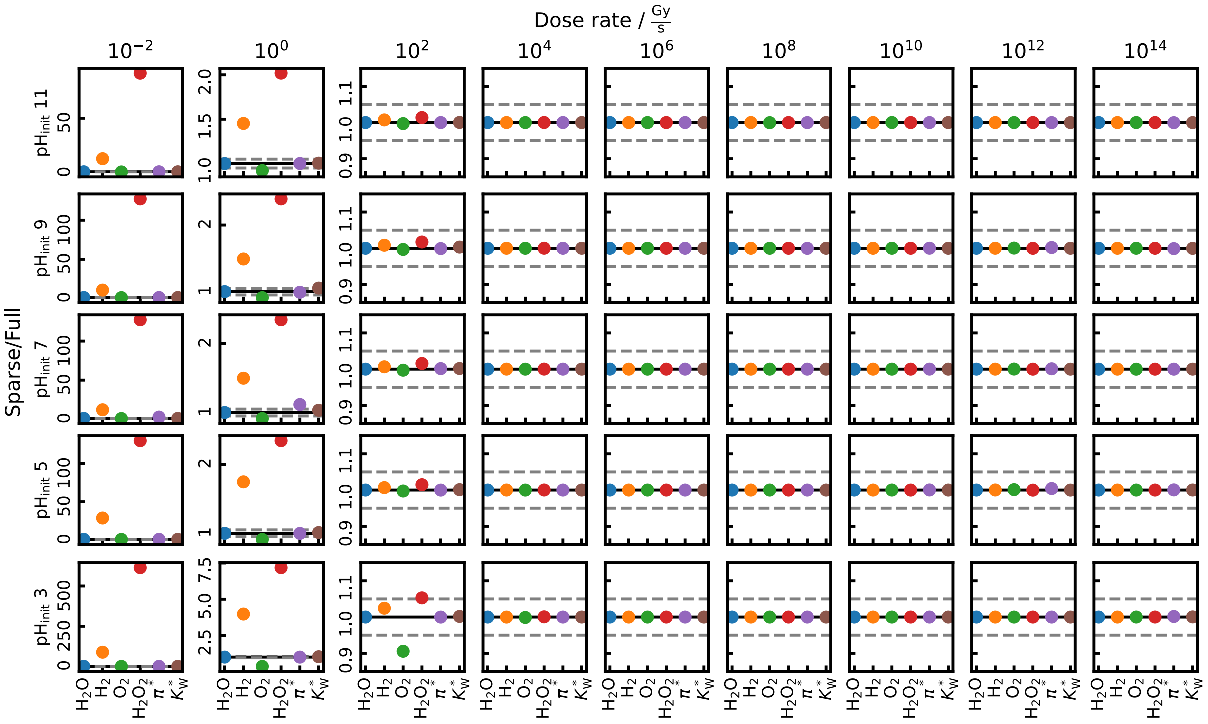

Figure S4: Excluded O.

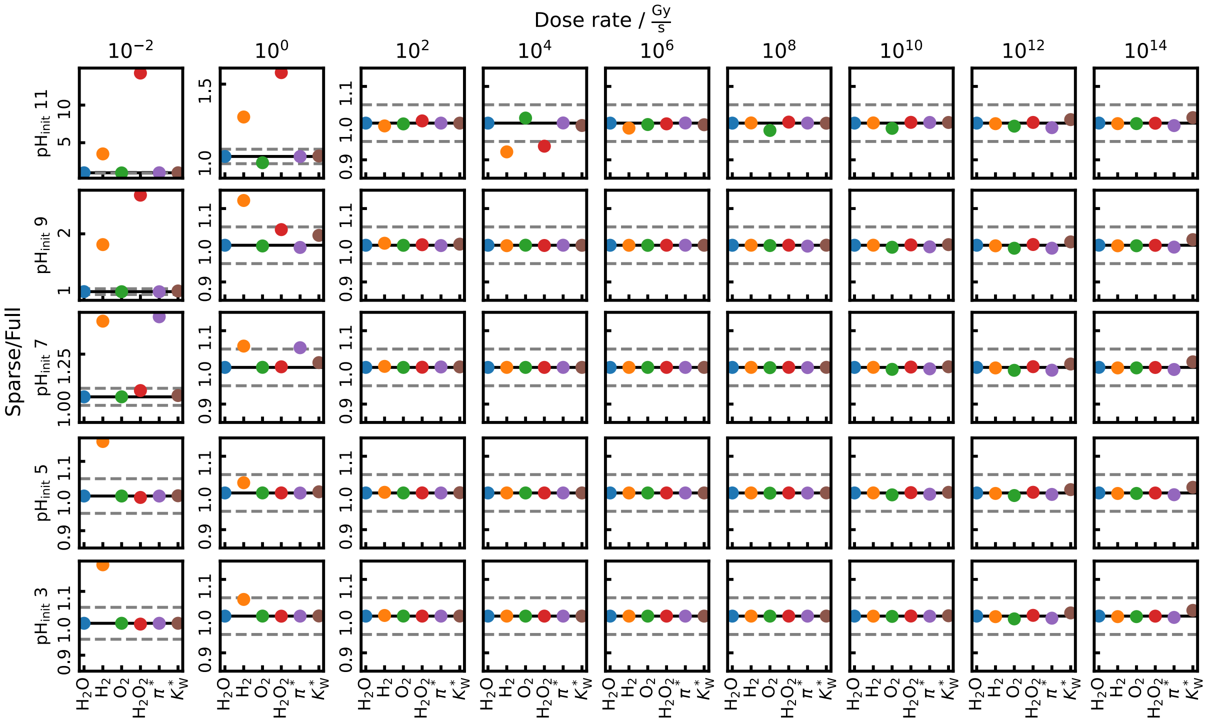

Figure S5: Excluded O^-, HO_3, O_3, and O_3^-

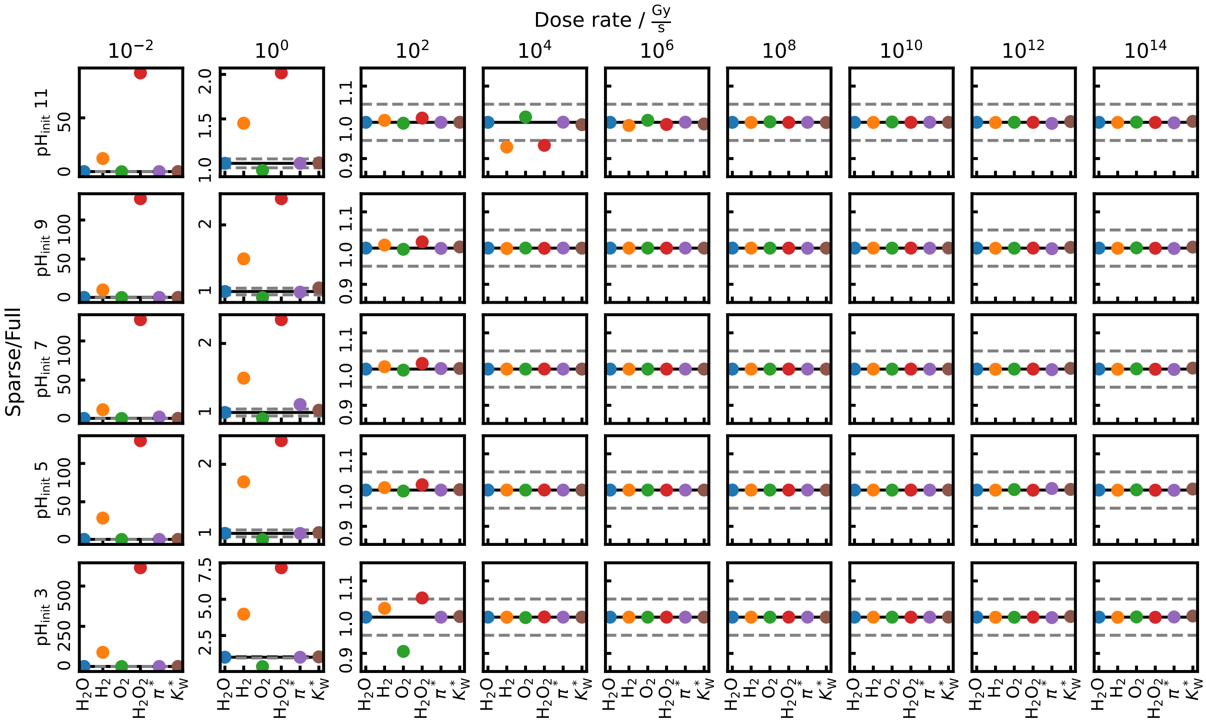

Figure S6: Excluded O, HO_3, O_3, and O_3^-

Additional spot checks for sparsening the $HAuCl_4$ set

Due to the large parameter space, here, not all possible combinations are tested. Instead, species are deactivated subsequently. Notably, not in all subsequent deactivations, all spot checks remain stable. Moreover, deactivation of a single species can lead to subsequent suppression of other reactants. Such cases are summarized in a single Figure. In addition, conditions not forming a steady state within 1000 s are omitted (this holds for example true for all Au-containing reactants).

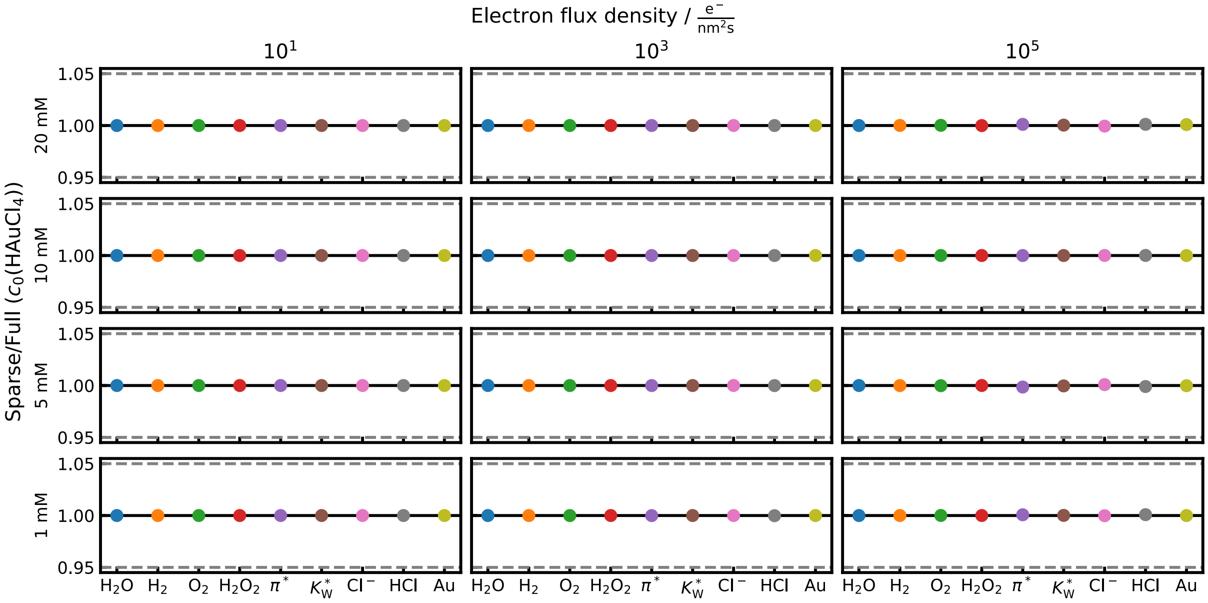

Figure S7: Excluded O_4.

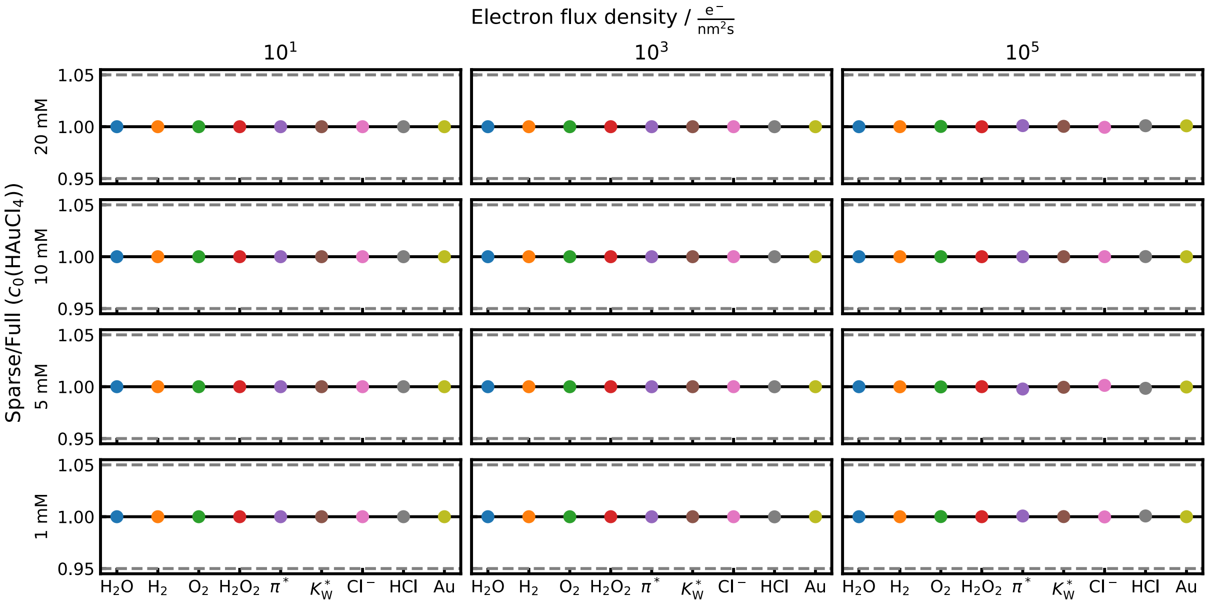

Figure S8: Excluded Cl_2O_4 and O_4.

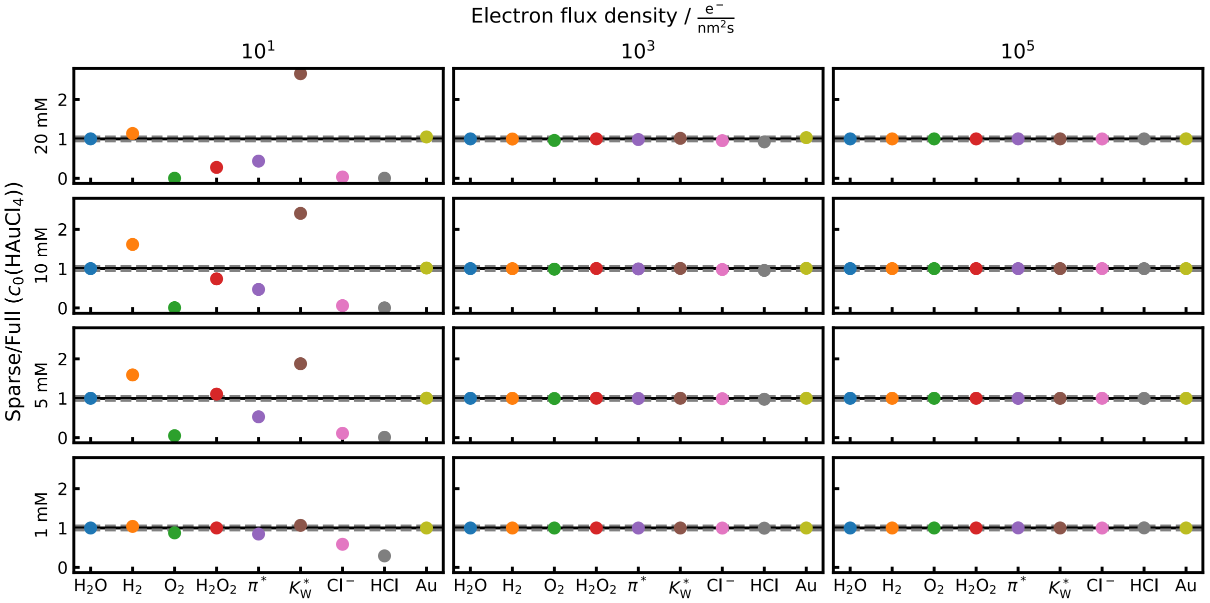

Figure S9: Excluded Cl_2O_2, Cl_2O_4, and O_4. This additionally deactivates Cl_2O. This spot check failed only at low electron flux densities. Thus, the procedure was continued.

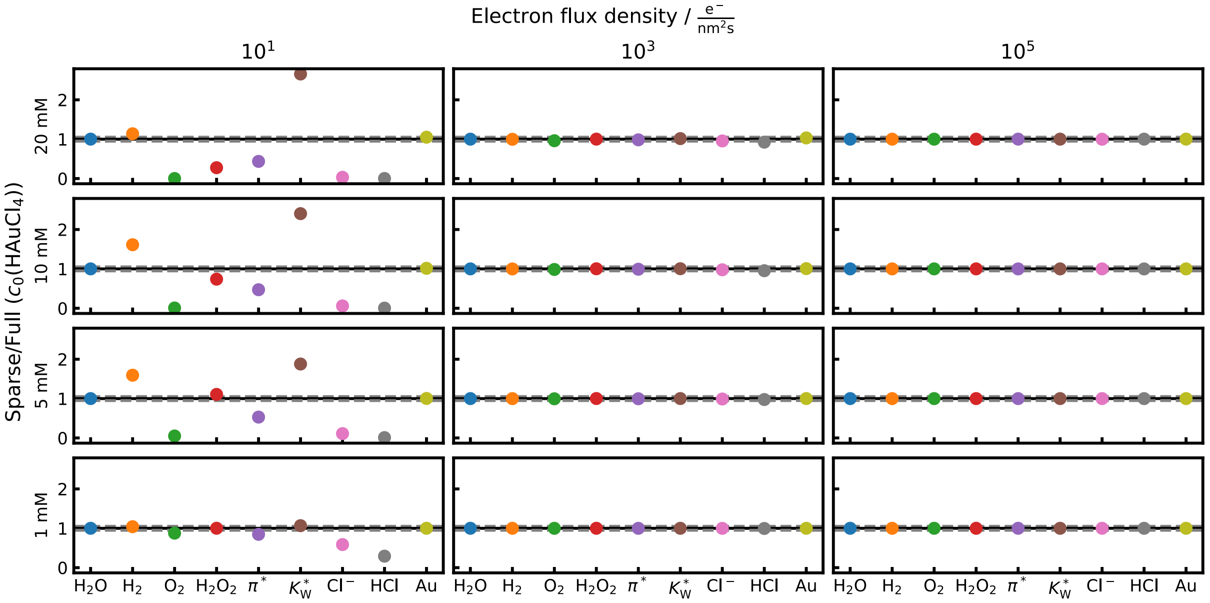

Figure S10: Excluded Cl_2O_3, ClO_3, ClO_3^-, Cl_2O, Cl_2O_2, Cl_2O_4, and O_4. This spot check failed only at low electron flux densities. Thus, the procedure was continued.

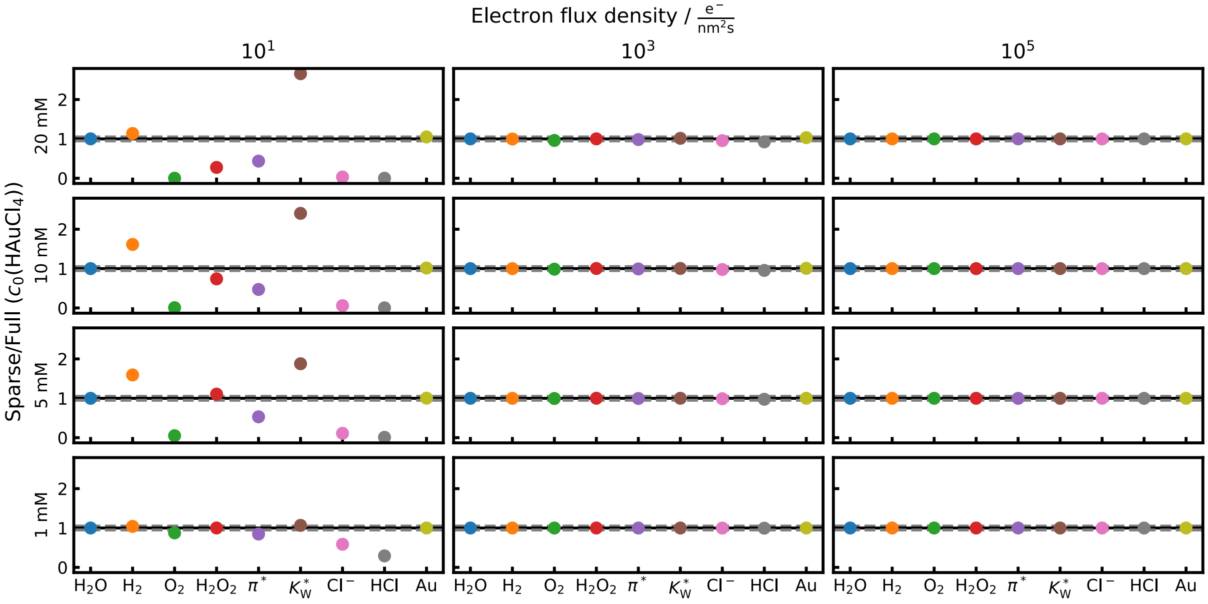

Figure S11: Excluded O, Cl_2O_3, ClO_3, ClO_3^-, Cl_2O, Cl_2O_2, Cl_2O_4, and O_4. This spot check failed only at low electron flux densities. Thus, the procedure was continued.

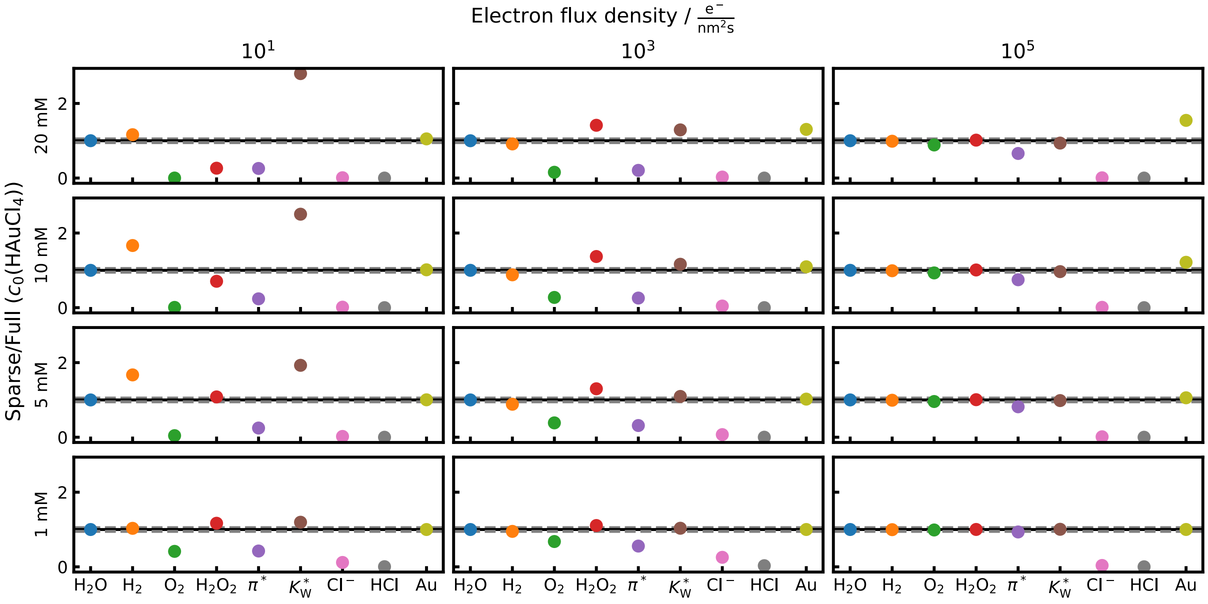

Figure S12: Excluded O^-, O, Cl_2O_3, ClO_3, ClO_3^-, Cl_2O, Cl_2O_2, Cl_2O_4, and O_4. This additionally deactivates HO_3, O_3, and O_3^-, as expected from the pure water study. This is considered as a failed test. Yet, the spot check can be restored by deactivating one additional reactant, as shown below.

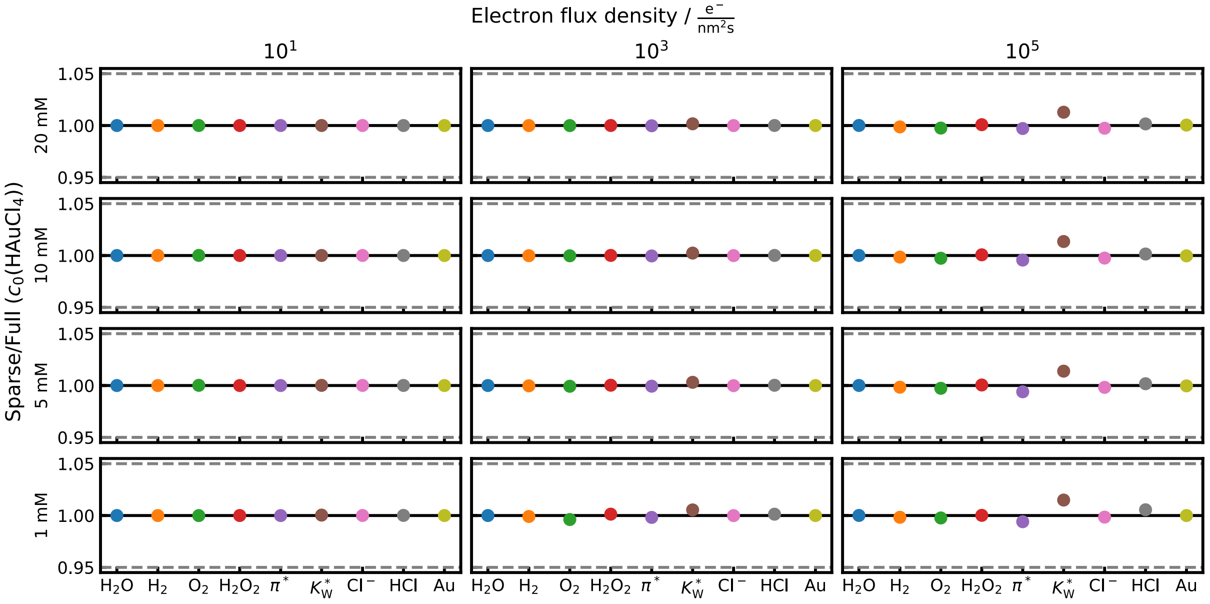

Figure S13: Excluded HClO, HO_3, O_3^-, O_3, O^-, O, Cl_2O_3, ClO_3, ClO_3^-, Cl_2O, Cl_2O_2, Cl_2O_4, and O_4. This additionally deactivates ClO, ClO_2, ClO_2^-, and Cl_2O_3.

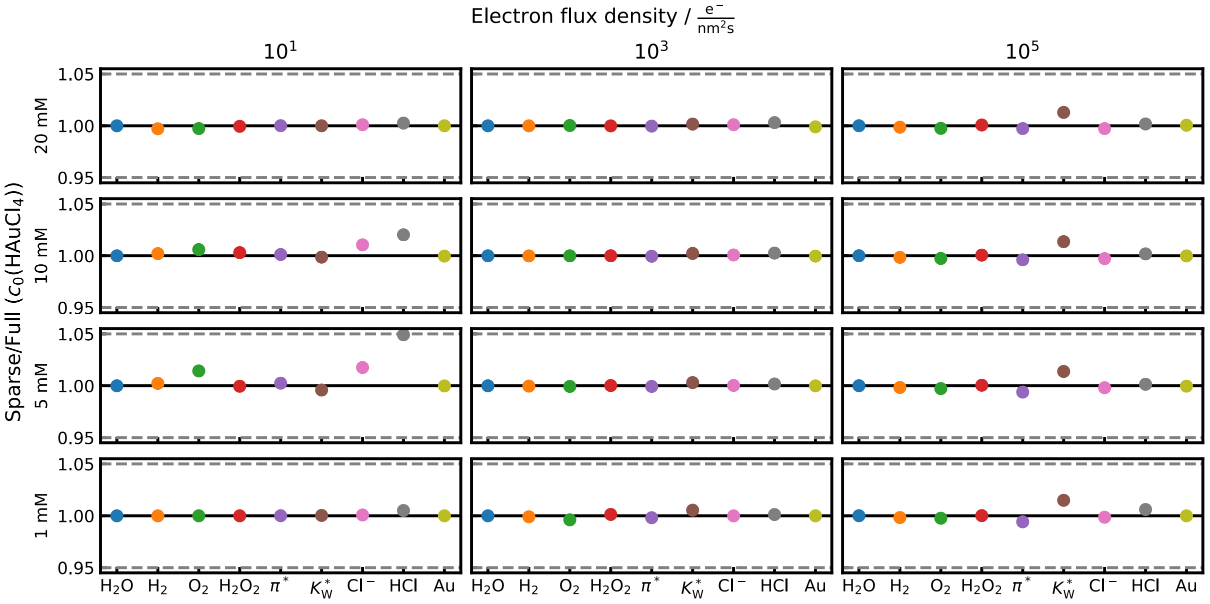

Figure S14: Excluded Cl_3^-, ClO, ClO_2, ClO_2^-, and Cl_2O_3, HClO, HO_3, O_3^-, O_3, O^-, O, Cl_2O_3, ClO_3, ClO_3^-, Cl_2O, Cl_2O_2, Cl_2O_4, and O_4.

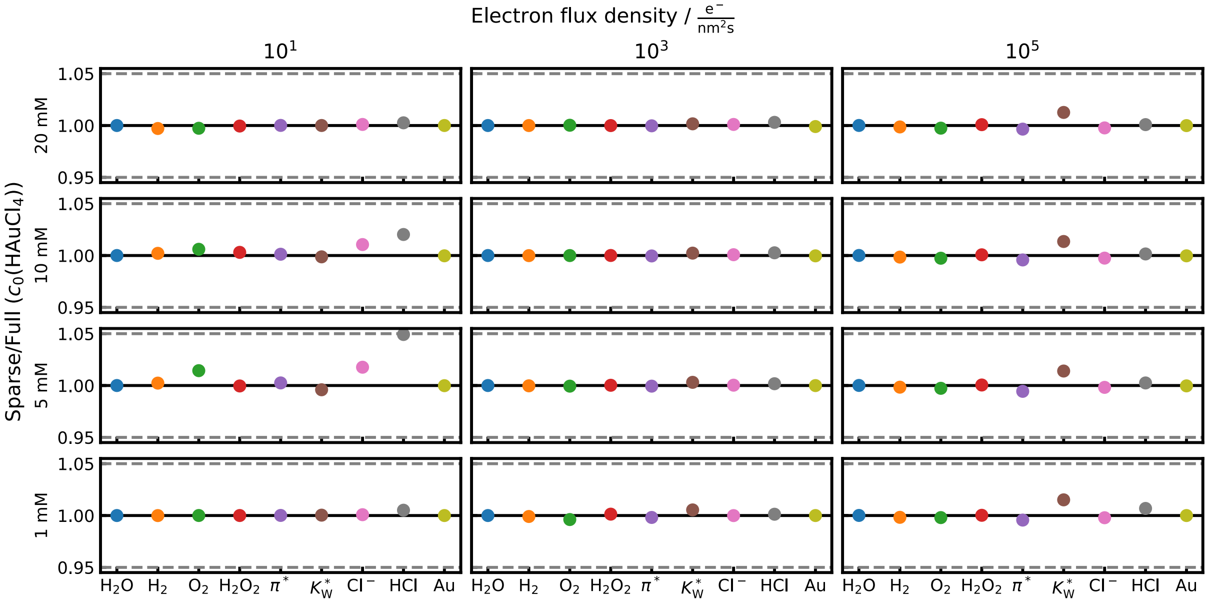

Figure S15: Excluded ClO^-, Cl_3^-, ClO, ClO_2, ClO_2^-, and Cl_2O_3, $HClO$, HO_3, O_3^-, O_3, O^-, O, Cl_2O_3, ClO_3, ClO_3^-, Cl_2O, Cl_2O_2, Cl_2O_4, and O_4.

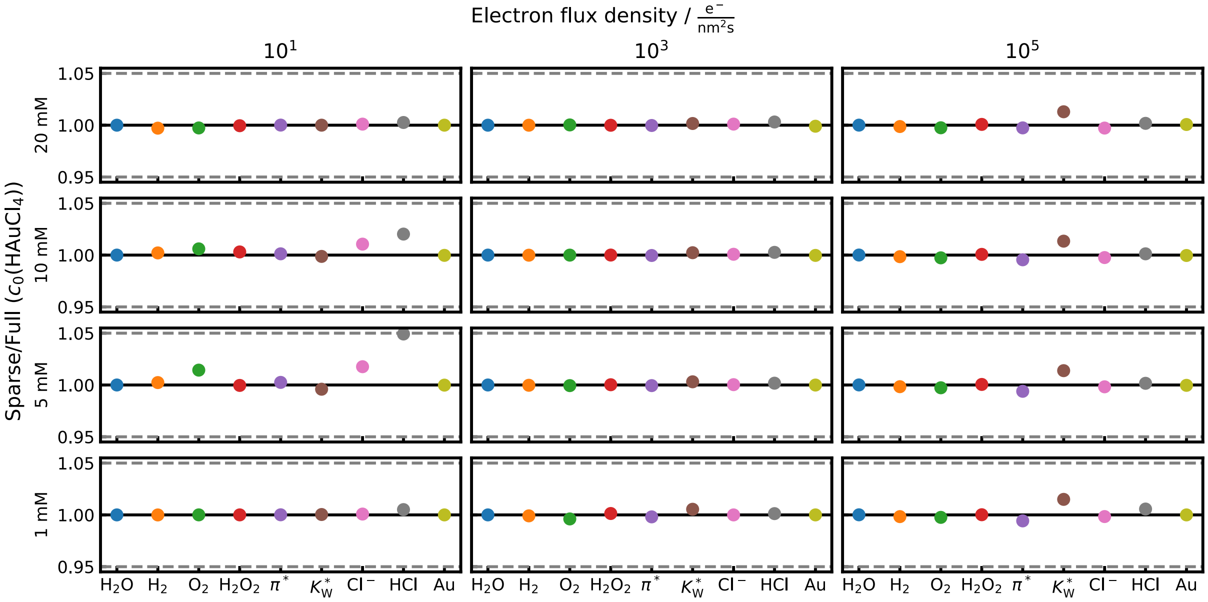

Figure S16: Excluded Cl_2, ClO^-, Cl_3^-, ClO, ClO_2, ClO_2^-, and Cl_2O_3, $HClO$, HO_3, O_3^-, O_3, O^-, O, Cl_2O_3, ClO_3, ClO_3^-, Cl_2O, Cl_2O_2, Cl_2O_4, and O_4. This figure displays the same data as Figure 4(a) in the main manuscript.

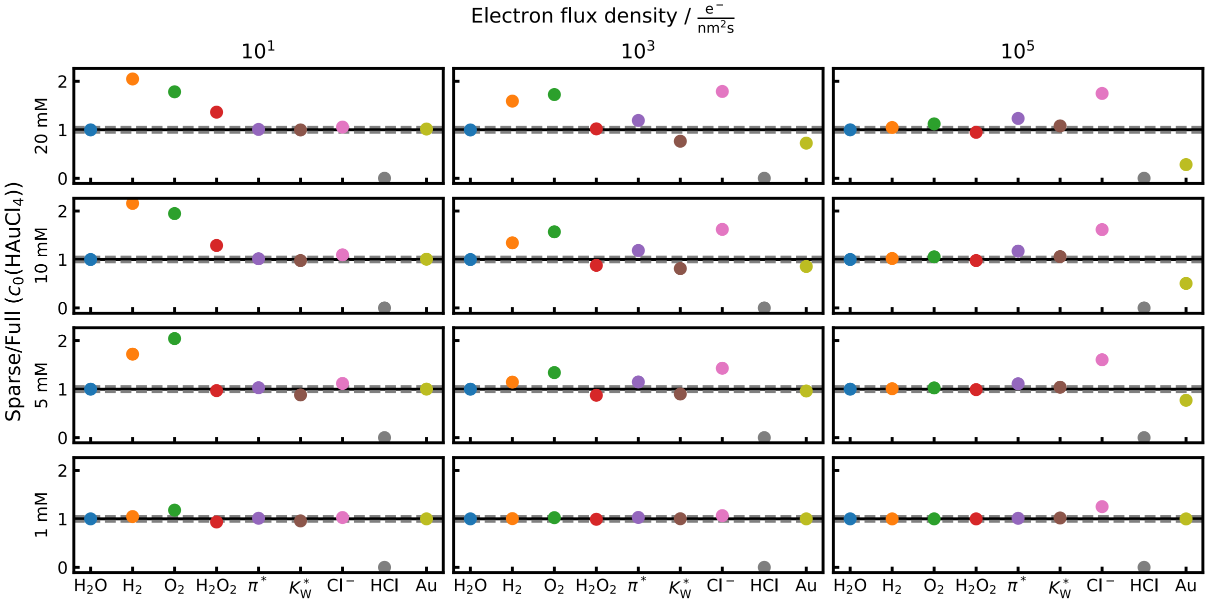

Figure S17: Failure after exclusion of Cl_2^- in addition to the situation shown in Figure 4(a) of the main manuscript.

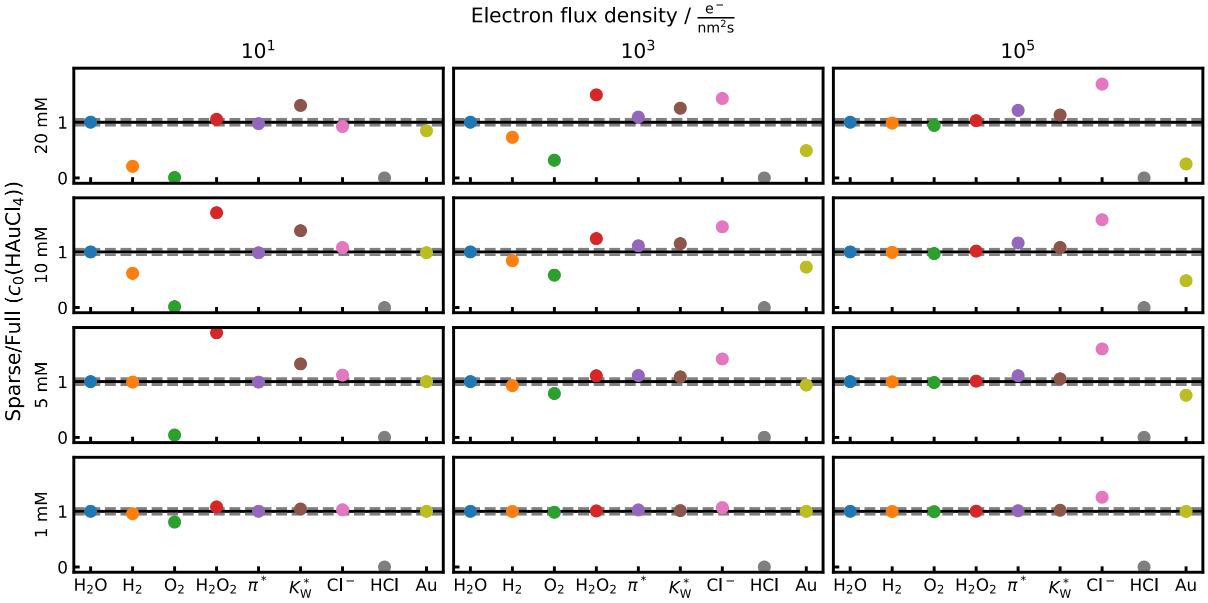

Figure S18: Failure after exclusion of Cl in addition to the situation shown in Figure 4(a) of the main manuscript.

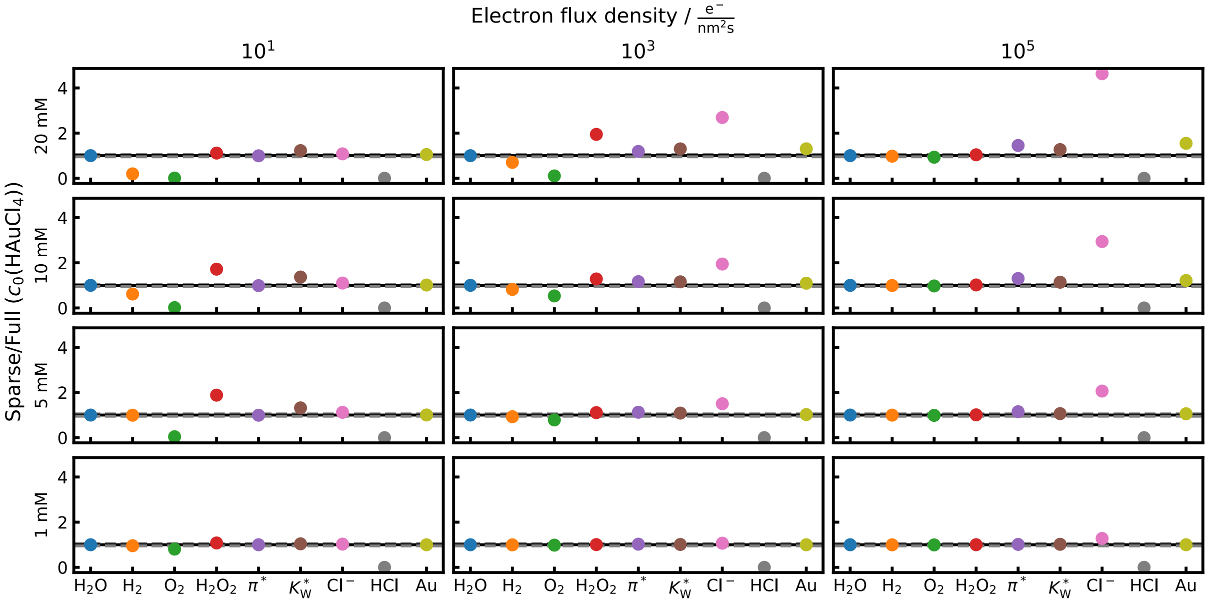

Figure S19: Failure after exclusion of $ClOH^-$ in addition to the situation shown in Figure 4(a) of the main manuscript. This deactivates Cl, as well.

Runtime analysis

To investigate the impact on runtime as an indicator on computational costs, the *timeit* module of Python was used to track the duration of the respective simulations. It is crucial to note that the simulation durations heavily depend on the input parameters such as dose rate, or initial concentrations. Therefore, the computation time was evaluated on the full text test matrices of either reaction set (see e.g., Figure 1, S1 – SS4 for the simulated parameter space in case of H_2O, and e.g., Figure 4(a), S5 – S17 for diluted $HAuCl_4$ solutions.).
This leads to 45 simulations per matrix for H_2O and twelve simulations per matrix for the $HAuCl_4$ case. To gain sufficient statistics, the simulation matrices were calculated three (ten) times for H_2O ($HAuCl_4$), yielding in total 135 (110) simulations. Moreover, to account for the dose rate-dependent differences in reaching a steady state[1,3], the simulated time interval varies between the H_2O and the $HAuCl_4$ model (10^6 s vs. 10^3 s). Therefore, the absolute computational durations are not comparable.

The tests were performed on a Lenovo ThinkPad (AMD Ryzen 7 PRO 5850U with Radeon Graphics (1.90 GHz), 16 GB RAM, Windows 10 Enterprise LTSC Version 21H2, 64bit x64-based processor). AuRaCh was executed using Python 3.10.6, NumPy 1.23.4, pandas 1.5.1, and SciPy 1.9.3. In both scenarios, the relative tolerance of the solver was set to 10^{-5}, whereas the absolute tolerance was set to 10^{-25} M.

The test results are denoted in Table S2 and visualized in Figure S20, showing a speed increase (gain) of about 3.7 for H_2O and 5.5 for $HAuCl_4$. The re-incorporation of O (see the following section) slows the simulation 1.3-fold. Note, that gains may differ for other ODE solvers.

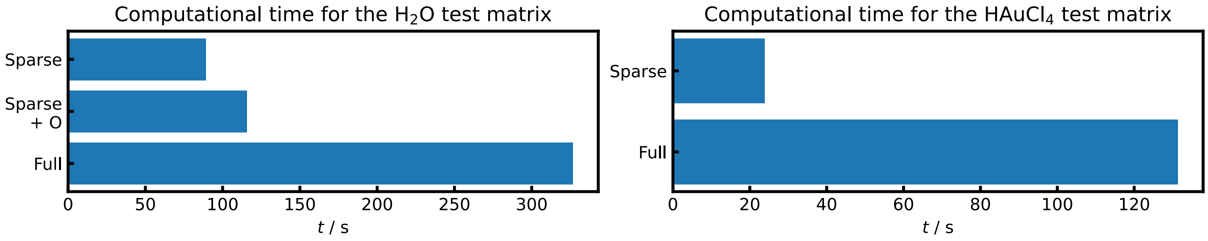

Figure S20:Illustration of the gain in sparsening for the kinetic models under investigation.

Table S2: Summary of the runtime analysis to estimate the gain in simulation speed. Note, that only the relative values (gain) are comparable between the discussed sets (sp = sparse).

<table>
<tr><th>Model</th><th>Total duration / s</th><th>Total duration / min</th><th>Avg. time per matrix / s</th><th>Avg. time per sim. / s</th><th colspan="2">Gain (Full/Sparse)</th></tr>
<tr><td>Full H_2O</td><td>980.128</td><td>16.335</td><td>326.709</td><td>7.260</td><td rowspan="2">3.671</td><td>n. a.</td></tr>
<tr><td>Sp. H_2O</td><td>276.007</td><td>4.450</td><td>89.002</td><td>1.978</td><td rowspan="2">1.300</td></tr>
<tr><td>Sp. H_2O + O</td><td>347.142</td><td>5.786</td><td>115.714</td><td>2.571</td><td>n. a.</td></tr>
<tr><td>Full $HAuCl_4$</td><td>1313.587</td><td>21.893</td><td>131.359</td><td>10.947</td><td colspan="2" rowspan="2">5.495</td></tr>
<tr><td>Sp. $HAuCl_4$</td><td>239.041</td><td>3.984</td><td>23.094</td><td>1.992</td></tr>
</table>

Investigations on steady-state decay

The sparsened set does not picture the decay of the steady states appropriately (c.f. Figure 6 in the main manuscript). However, it is worth noting that this is neither possible with the set used by Schneider *et al.*, as they purposely omitted long-term decay pathways[4]. This is illustrated in the Supporting Figure S21.

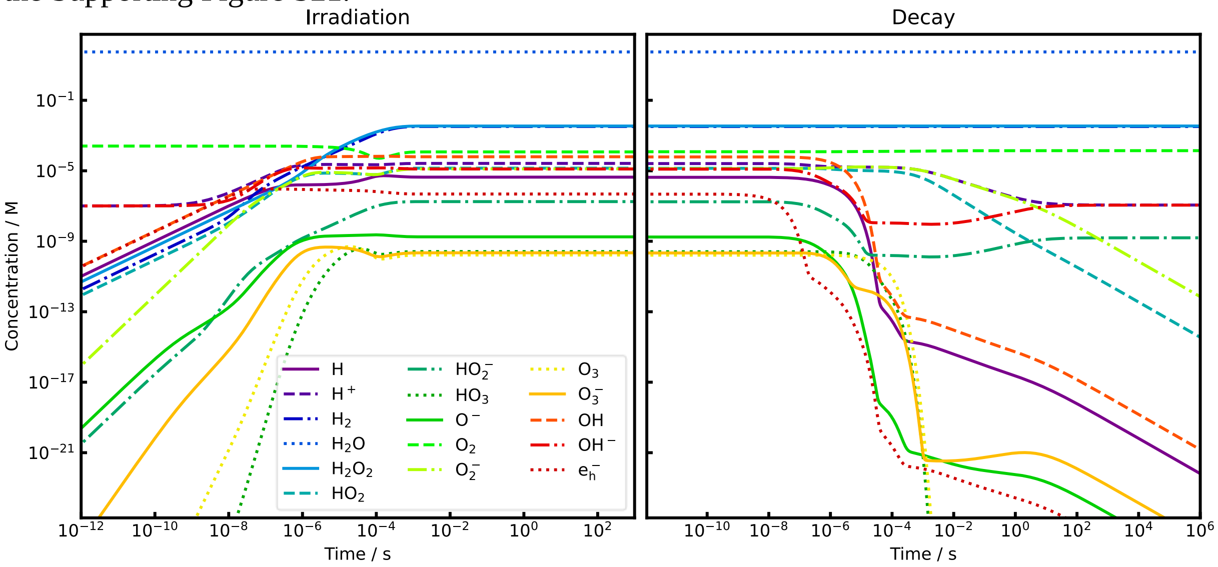

Figure S21: Steady state formation and decay of the reaction set including only the reactions regarded by Schneider et al.[4] at similar conditions as the data shown in Figure 6 of the main manuscript.

To potentially identify a model that could do so, it is insufficient to rely on a single number per constraint, as it can be done for steady state concentrations. Instead, both, slope, and actual values of the decay of the experimentally accessible parameters must be maintained. Hence, the full data range should be analyzed.

As possibility to quantify the agreement between the data of a comprehensive and a sparsened model, we define a metric that we call *goodness of agreement* Δ. Inspired by the well-known goodness of fit metric *coefficient of determination* (R^2), it is defined as follows:

$$\Delta = 1 - \frac{\sum(y - f)^2}{\sum(y - \bar{y})^2} \qquad (S1)$$

Here, y describes the time-dependent data of a decay curve in the comprehensive set (with $\bar{y}$ as mean value), whereas f stands for the sparsened reaction set. The similarity to the definition of R^2 becomes obvious when interpreting y as the "true" (i.e., measured, experimental) data that is supposed to be fitted/modeled by f. Consequently, a value of 1 describes perfect agreement over all time points. This is tested for the sparsened reaction set including O which is characterized for steady state formation in Figure S5 (see above), as well as additionally labeled in Table S1.

The results for the parameter space for pure water are shown in Figure S21. Exemplary decay simulations can be found in Figure 6 in the main manuscript.

Evidently, the agreement between this semi-sparsened set and the comprehensive model is not matching our findings for steady state formation. We attribute this to the more-sophisticated comparison method, as here, transients and not steady state values must be compared. Nevertheless, the decay at medium-to-high dose rates is well reflected by this approach. It is also notable that it is in better agreement at low than at high initial pH. We speculate that this may be related to anionic decay pathways during high initial pH, e.g., via O^-. Moreover, we note that at extremely high dose rates and medium initial pH, this model shows drastic deviations for π^*. However, as discussed in the main manuscript, the model fails to precisely capture physical processes at such intensive radiation anyway so that these values are of minor importance.

Nonetheless, adding only O to the sparsened reaction set facilitates transient analyses in a large parameter space that is of utmost relevance to LP-TEM.

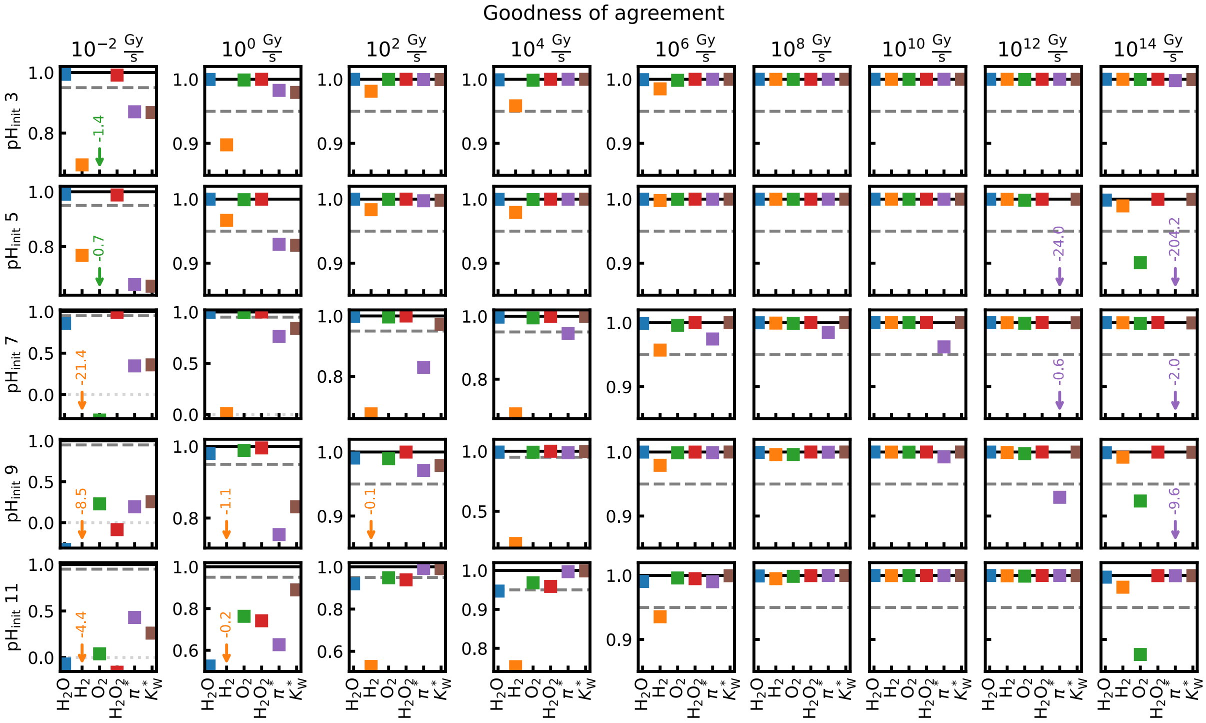

Figure S22: Goodness of agreement after eq. (S1) for the sparse reaction set including O during steady state decay. The dashed lines denote 0.95. To visualize small changes within well described metrics, some drastic outliers are indicated by respective annotations.

Additional G-value test

Table S3: Generation values for low-LET radiation (i.e. γ- or hard X-rays) after Pastina and LaVerne[5].

Reactant	e_h^-	H^+	OH^-	H_2O_2	H	OH	HO_2	H_2	H_2O
$G_i / \frac{\text{Molecules}}{100\text{ eV}}$	2.60	3.10	0.50	0.70	0.66	2.70	0.02	0.45	-4.64

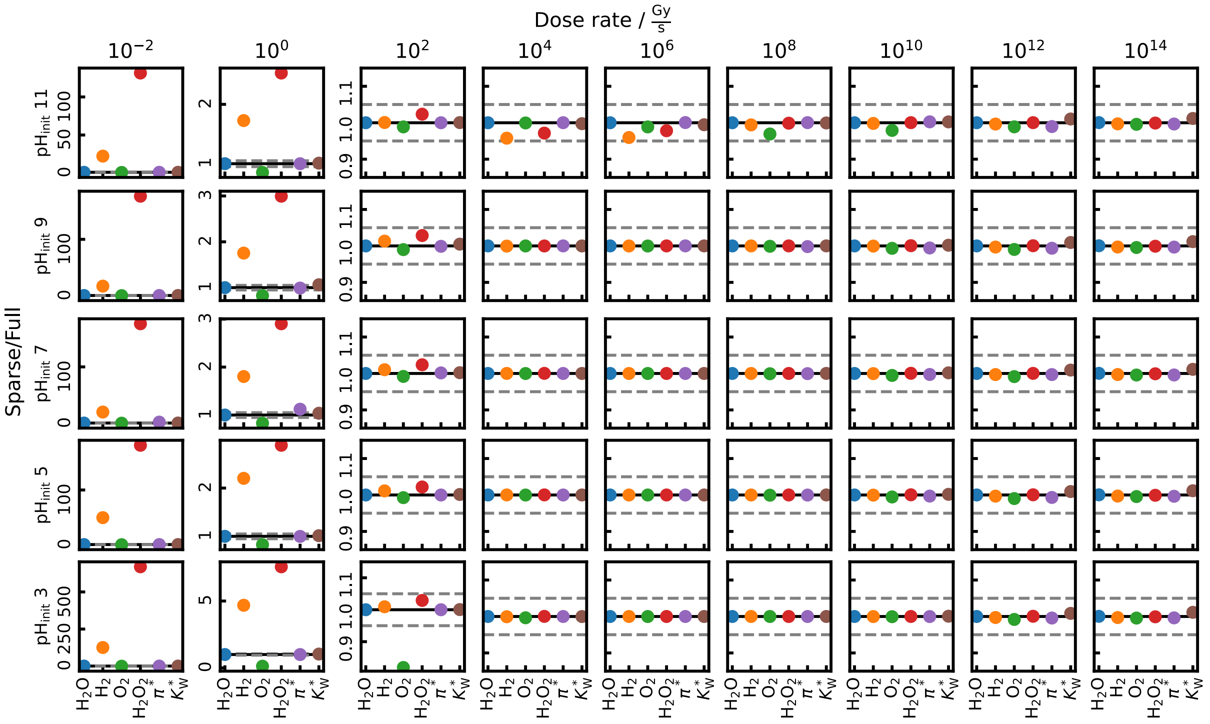

Figure S23: Relative deviation of the testing parameters of pure water sparsening down the kinetic model to twelve reactants using the G-values listed in Table S3. The solid line denotes a perfect agreement between the full and the sparsened model, whereas the dashed lines mark the 5% accuracy limit.

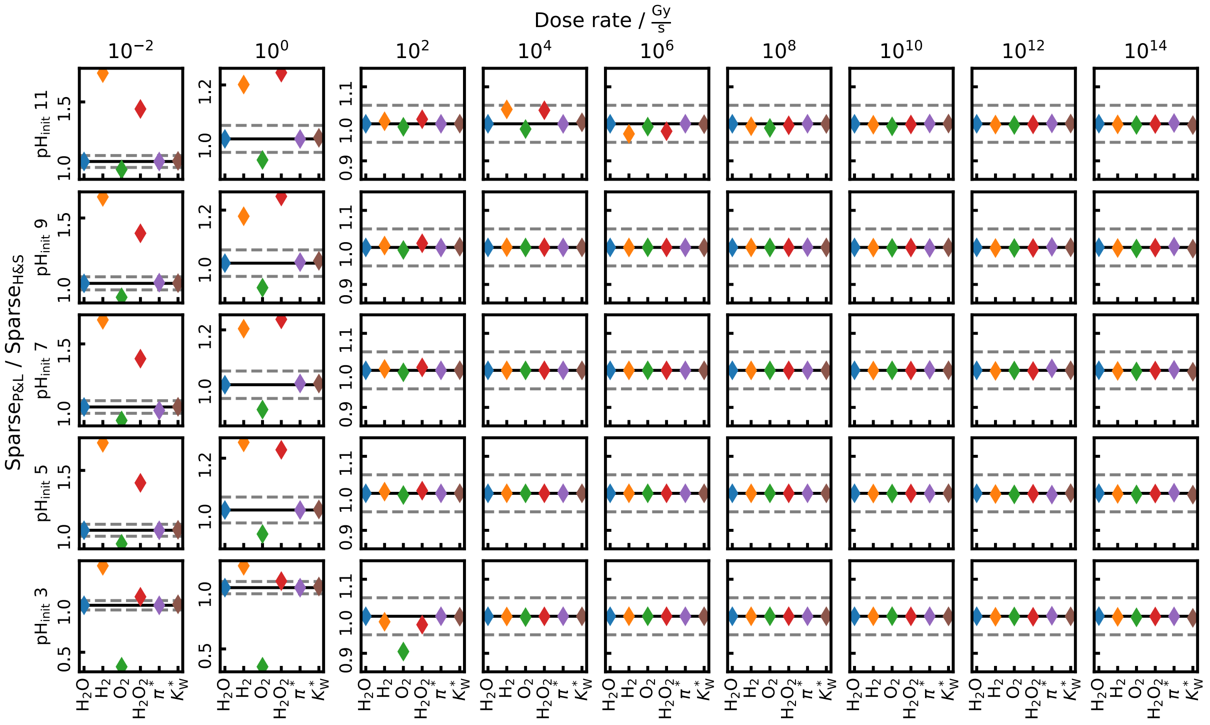

Figure S24: Relative deviations in the testing parameters for pure water caused by using the sparsened water model with the G-values of Pastina and LaVerne (P&L, see Table S3) compared toγ the G-Values proposed by Hill & Smith (H&S, see Table 1). The respective absolute values are shown in Figure 2 (H&S) and S19 (P&L). The dashed lines denote ±5%.

Supporting References

(1) Fritsch, B.; Zech, T. S.; Bruns, M. P.; Körner, A.; Khadivianazar, S.; Wu, M.; Zargar Talebi, N.; Virtanen, S.; Unruh, T.; Jank, M. P. M.; Spiecker, E.; Hutzler, A. Radiolysis ‐ Driven Evolution of Gold Nanostructures ‐ Model Verification by Scale Bridging in situ Liquid ‐ Phase Transmission Electron Microscopy and X ‐ Ray Diffraction. *Advanced Science* **2022**, *9* (25), 2202803. DOI: 10.1002/advs.202202803.

(2) Hill, M. A.; Smith, F. A. Calculation of initial and primary yields in the radiolysis of water. *Radiation Physics and Chemistry* **1994**, *43* (3), 265–280. DOI: 10.1016/0969-806X(94)90190-2.

(3) Fritsch, B.; Körner, A.; Couasnon, T.; Blukis, R.; Taherkhani, M.; Benning, L. G.; Jank, M. P. M.; Spiecker, E.; Hutzler, A. Tailoring the Acidity of Liquid Media with Ionizing Radiation: Rethinking the Acid-Base Correlation beyond pH. *The Journal of Physical Chemistry Letters* **2023**, *14* (20), 4644–4651. DOI: 10.1021/acs.jpclett.3c00593.

(4) Schneider, N. M.; Norton, M. M.; Mendel, B. J.; Grogan, J. M.; Ross, F. M.; Bau, H. H. Electron–Water Interactions and Implications for Liquid Cell Electron Microscopy. *The Journal of Physical Chemistry C* **2014**, *118* (38), 22373–22382. DOI: 10.1021/jp507400n.

(5) Pastina, B.; LaVerne, J. A. Effect of Molecular Hydrogen on Hydrogen Peroxide in Water Radiolysis. *The Journal of Physical Chemistry A* **2001**, *105* (40), 9316–9322. DOI: 10.1021/jp012245j.
